# Role of the gut microbiota in the pathogenesis of coeliac disease and potential therapeutic implications

**DOI:** 10.1007/s00394-020-02324-y

**Published:** 2020-07-10

**Authors:** Anthony K. Akobeng, Parul Singh, Manoj Kumar, Souhaila Al Khodor

**Affiliations:** 1 Division of Gastroenterology, Hepatology, and Nutrition, Sidra Medicine, Doha, Qatar; 2 Weill Cornell Medicine, Cornell University, Doha, Qatar; 3 Research Department, Sidra Medicine, Doha, Qatar

**Keywords:** Coeliac disease, Microbiota, Metagenomics, Dysbiosis

## Abstract

**Purpose:**

Although genetic predisposition and exposure to dietary gluten are considered necessary triggers for the development of coeliac disease, alterations in the gut microbial composition may also contribute towards the pathogenesis of coeliac disease. This review aims to provide an overview of the available data on the potential mechanisms through which the gut microbiota plays a role in the causation of coeliac disease and to discuss the potential therapeutic strategies that could diminish the consequences of microbial dysbiosis.

**Method:**

A search of the literature was performed using the PubMed, Embase, and JSTOR databases; relevant articles were included.

**Results:**

Recent studies in patients with coeliac disease have reported an increase in the relative amounts of gram negative bacterial genera such as *Bacteroides*, *Prevotella*, and *Escherichia*, and reduced amounts of protective anti-inflammatory bacteria such as *Bifidobacteria* and *Lactobacilli*. Dysbiotic microbiota may lead to a dysregulated immune response that may contribute to the pathogenesis of coeliac disease. In infancy, antibiotic use and certain infant feeding practices may lead to alterations in the developing gut microbiota to influence the immune maturation process and predispose to coeliac disease.

**Conclusion:**

The induction of the intestinal immune system and gluten intolerance may be influenced by the relative abundance of certain microbiota. Factors such as infant feeding practices, diet, antibiotics, and infections, may be involved in the development of coeliac disease due to their influence on gut microbial composition. The efficacy of potential modulators of the gut microbiota such as probiotics, prebiotics, and fecal microbial transplant as adjunctive treatments to gluten-free diet in coeliac disease is unproven and requires further investigation.

## Introduction

Coeliac disease is an autoimmune disorder triggered by the ingestion of gluten in genetically susceptible people [1]. The disorder is characterized by a mucosal disease of the proximal small bowel as a result of a T-cell mediated destruction of mucosal epithelial cells. It is generally acknowledged that coeliac disease affects about 1% of the population with an increasing prevalence [2] that varies between countries [3]. Patients with coeliac disease develop a permanent loss of immune tolerance to gluten [4, 5], a protein found in cereals such as wheat, rye, and barley. Upon ingestion, gluten can cause a pathological injury characterized by progressive degrees of inflammation and loss of villi in the proximal small bowel leading to the development of gastrointestinal malabsorption along with extra-gastrointestinal manifestations [3].

Coeliac disease is a multifactorial disease, characterized by a complex interplay of genetic and environmental factors. While genetic factors (such as the presence of Human Leukocytic Antigen—mainly HLA-DQ2 or HLA DQ-8) and exposure to dietary gluten are considered to be necessary triggers, they are not sufficient for disease development [6]. Additional factors such as infant feeding practices, the amount of gluten ingested, the age at which gluten is introduced, neonatal infections, rotavirus infections, and early life exposure to antibiotics can play a pathogenic role in the development of the disease possibly through modulation of the gut microbiota [7–9].

The human gastrointestinal tract harbors approximately 100 trillion micro-organisms—mainly bacteria, but also viruses, fungi, and protozoa [10, 11]. The gut microbiota provides the host with several significant benefits including maintaining the integrity of the mucosal barrier, providing nutrients such as vitamins, protecting against pathogens, and helping to regulate immune function [12]. Various chronic disorders such as inflammatory bowel disease [13], and type 1 diabetes [14] have been shown to be associated with alterations in the gut microbial composition. Recent studies have also suggested that intestinal dysbiosis may play a role in the pathogenesis of coeliac disease [15–18].

In this article, we aim to discuss the latest findings regarding the intestinal microbiota composition, and its potential mechanisms for causing coeliac disease. We also highlight the current knowledge on the enteric virome and the mobile microbiota such as oral and blood microbiota in relation to coeliac disease. Finally, we discuss the therapeutic strategies that could diminish the consequences of microbial dysbiosis in coeliac disease.

## Methods

A medical literature search was conducted using PubMed, Embase, and JSTOR databases for articles published from the beginning of the database until Jan 5th, 2020. The initial search was done using the general search terms: (coeliac disease OR celiac disease OR gluten-sensitive enteropathy) AND (gut microbiota OR gut microbiome OR intestinal microbiome). Additional searches were performed to collect specific data on the use of diet, probiotics, prebiotics, antibiotics, and fecal microbial transplantation in coeliac disease. Only articles published in English were included. No restrictions were placed on the study design or the type of article. References of the included articles were also reviewed for additional relevant articles.

## Results

**Fig. 1:**
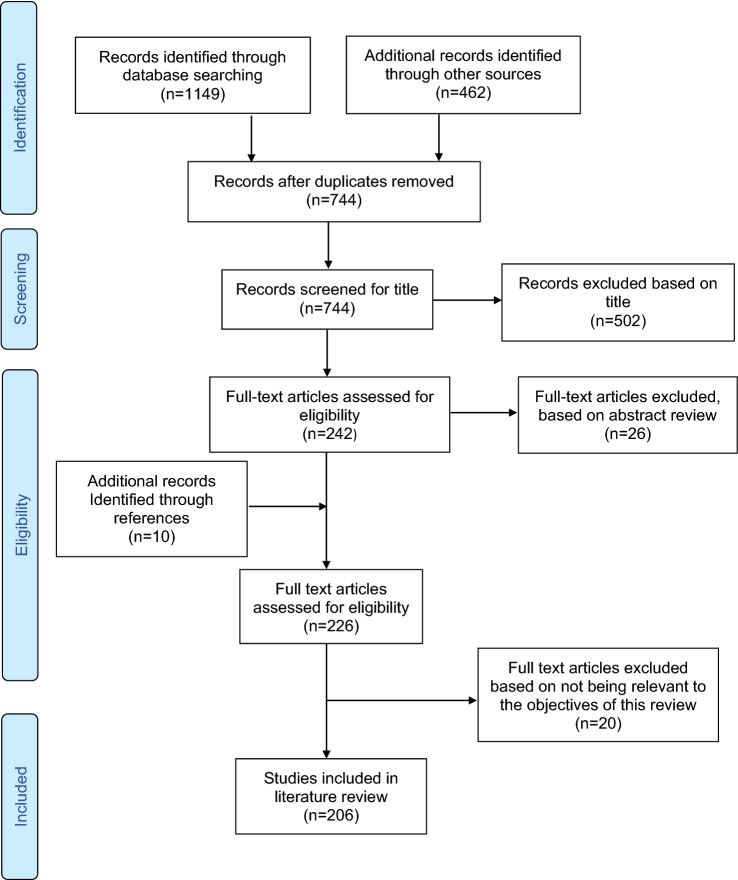
Flowchart of the Study selection for literature review

Figure 1 details the flow-chart of the literature review process. We have included 206 studies based on their relevance to the review topic.

## Gluten and immune system in coeliac disease

Several studies have highlighted the complex interplay between host polygenic disorder and the ingested gluten proteins in disturbing the host epithelial functions in patients with coeliac disease [19–21], but little is known about the multifactorial interplay and how gluten starts the entire process.

The primary event of a gluten-induced inflammatory immune response requires that gluten peptides translocate via intracellular or paracellular mechanisms through the intestinal epithelial barrier [22] and have access to the lamina propria. Glutens consists of the subunits of monomeric gliadins and polymeric glutenins. The high content of proline and glutamine-rich polypeptide residues in the gluten subunits makes them resistant to the proteolytic degradation by the gastrointestinal enzymes, thus, yielding rather large peptides, up to 33-mer in length [23]. Intestinal epithelia under normal physiological conditions are impermeable to macromolecules, such as gliadin. In coeliac disease, the integrity of the tight junction (TJ) system in the epithelial cells layer is compromised partly due to the upregulation of zonulin, an intestinal peptide involved in TJ regulation [24]. Nonetheless, the relatively poor digestion of these proteins or the resulting increased gut permeability is not sufficient to cause coeliac disease [25, 26]. The multifactorial pathogenesis of coeliac disease appears to stem from genetic susceptibility which, in addition to other unknown contributing factors, triggers the innate and adaptive immune response. In a process called deamidation, tissue transglutaminase 2 (tTG2) modifies gluten peptides to negatively charged glutamic acid [27]. The deamidated gluten peptides in the lamina propria are then taken up by the dendritic cells (DC) carrying human leukocyte antigen (HLA-DQ2 and HLA-DQ8) haplotypes [21], which in turn modulate the gluten-specific immune response by activating the gluten-reactive CD4 + T cells and intraepithelial-CTLs [21, 22]. Different types of HLA complexes such as HLA-DQ2.5 (HLA-DQA1*05/HLA-DQB1*02), HLA-DQ8 (HLA-DQA1*03/HLA-DQB1*03:02), and HLADQ2.2 (HLA-DQA1*02:01/HLA-DQB1*02) are associated with coeliac disease pathogenesis [28, 29] and have been reported in more than 90% of coeliac disease patients [30].

**Fig. 2:**
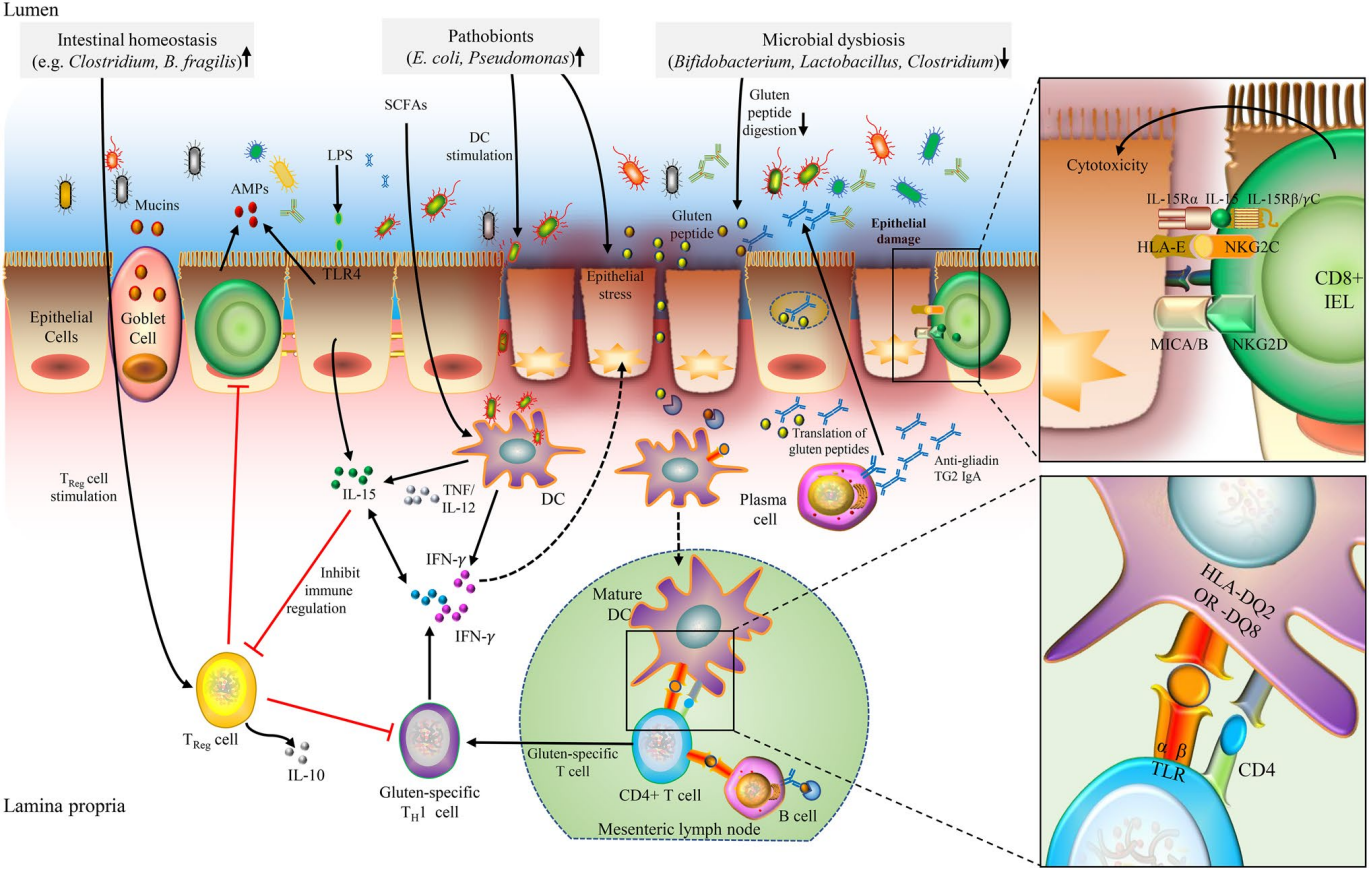
The immune response in coeliac disease pathogenesis is mediated by both B cell and T cell response. The intestinal microbiota, both commensals microbes and pathobionts, might contribute to the development of coeliac disease by influencing the gluten peptide digestion, stimulation of DC and T_Reg_ cells, epithelial cell stress, intestinal permeability modulation, and pro-inflammatory cytokines production. Microbial flagellin or LPS can stimulate AMPs from epithelial cells and release of mucins from goblet cells. The translocated gluten peptides in the lumen of the small intestine are first deamidated by the tissue TG2 in the lamina propria and are then taken up by the dendritic cells to trigger the proinflammatory gluten-specific CD4 + T-cell response in the mesenteric lymph nodes. The activated CD4 + T-cell prime the B-cells to produce different types of antibodies such as anti-gliadin, anti-TG2 IgA antibodies and also stimulate the gluten-specific TH1 cell to produce IL-21 and IFN-γ cytokines to cause intestinal inflammation in genetically predisposed hosts. Activation of the innate immune response is a key initial step in coeliac disease. Increased epithelial stress due to microbial dysbiosis or ingested gluten peptides can trigger stress molecules on epithelial cells (HLA-E, MICA/B) and induce IL-15 production from epithelial cells. In the presence of IL-15, CD8 + IELs can induce epithelial lesions via production of different cytotoxic molecules (perforin or granzymes). *DC* dendritic cells, T_Reg_ cells T Regulatory cells, *IL* interleukin, *TG2* transglutaminase 2, *IgA* immunoglobulin A, *IFN* interferon, *LPS* lipopolysaccharides, *SCFAs* short-chain fatty acids, *TNF* tumor necrosis factor, *Th* T helper, *HLA* human leukocyte antigen, *TLR* toll-like receptors, *IEL* intraepithelial lymphocytes, *NKG2C* natural killer group 2C receptor, *NKG2D* natural killer group 2D receptor

Stimulated gluten-specific CD4 + T cells can result in the overactivation of the various immune functions—for example activated CD4 + T cells can further stimulate B cells to produce autoantibodies such as anti-gluten, transglutaminase 2 (TG2) and anti-tissue antibodies [22, 31] that could induce changes in the cytoskeleton of enterocyte through actin redistribution and the consequent epithelial cell damage [32]. The CD4 + T cells can also produce a high level of pro-inflammatory cytokines and induce specific T_H_1 cells leading to the production of excessive amounts of IL-21 and IFN-γ [33]. As a result, the undue amounts of IL-21 and IFN-γ in the lamina propria not only exacerbate intestinal inflammation, epithelial tissue damage and tissue junction disassembly, but also lead to fibroblast or lamina propria mononuclear cell secretion of matrix metalloproteinases. These are responsible for tissue remodeling resulting in villus atrophy and crypt hyperplasia, the known characteristics of coeliac disease (Fig. 2). Intriguingly, the increased production of IL-15 by intestinal epithelial cells was observed only in coeliac disease patients with HLA-DQ allotypes, not only disturbing the integrity of the epithelial barrier to induce coeliac disease pathogenesis, but also altering the intestinal immune regulation [34]. For example, IL-15 has been found to inhibit the inducible differentiation of T_Reg_ cells [35], induce cytotoxic killing of epithelial cells [36, 37], and trigger the production of IL-21 [38] leading to increased epithelial transport of toxic and immunogenic gluten peptides in coeliac patients [39]. Furthermore, it has been shown that IL-15 stimulates the activation of IFN-γ producing CD8 + IELs (intraepithelial lymphocytes) such as (CD8 + TCRαβ + and TCRγδ + T cells harboring the NK receptor) in the lamina propria, and contributes to the selective expansion of the IEL population independently of antigen presentation [30], indicating the crucial role of IL-15 in causing inflammation and lesions in the intestines.

It is currently unclear how gluten could have such a range of immunological effects (as shown above) and how it binds to unrelated receptors. Further studies are needed to identify the exact molecular mechanisms involved in the immunogenicity of gluten. Based on the current data, it is fair to conclude that in genetically susceptible individuals, gluten leads to the activation of cellular stress pathways and the release of proinflammatory mediators promoting the development of inflammatory T-cell responses. Clinical trials of drugs aimed at reducing the immune activation in response to gluten are currently underway with promising results [40].

## Gut microbiota and coeliac disease

Approximately 30% of the general population bears the main susceptibility genes for coeliac disease (HLA-DQ2 and HLA DQ-8), but only a small proportion of these individuals can develop the disease [41]. Studies have shown, in contrast to the original hypothesis, that coeliac disease can occur at any age [42] irrespective of delayed or early gluten introduction [9, 43], and in some cases years after the introduction of gluten [9]. The increasing number of patients experiencing the onset of coeliac disease in adult life despite their early exposure to gluten [44], together with the lack of 100% concordance of coeliac disease in monozygotic twins, suggests that additional factors such as infections and changes in the gut microbial composition may contribute to pathogenesis and full manifestation of the disease [45]. The intestinal microbiota establishes mutual relationships with the human host and plays a central role in the host immune system regulation. Healthy gut microbiota supports many aspects of overall health while several complex inflammatory immune-mediated diseases including IBD [46], obesity, and diabetes [47, 48] have been linked to dysbiosis of the gut microbiota.

### The human gut microbiota

The human microbiota consists of a wide range of bacteria, viruses, fungi, and archaea that live inside and on our bodies. The largest population of microbes resides in our gut and consists of 70% of all microbes [49]. It is estimated that we can bear up to 2 kg of microbes in our gut, and out of those trillions of microorganisms there are at least 500–1000 species of bacteria with more than 3 million genes overwhelmingly exceeding the 20,000–25,000 human genes [50–52]. *Firmicutes* (60–80%) and *Bacteroidetes* (15–25%) are the dominant bacterial phyla, which along with other less abundant phyla such as *Proteobacteria*, *Actinobacteria* and *Verrucomicrobia* make up 98% of the gut microbiome in healthy adults. The microbial species diversity and density increase distally from the stomach to the colon, which contains the highest microbial density recorded in any habitat on Earth with over 10^13^ microbial cells [53]. The initial colonization of the gut by microbes is one of the most important processes in human life. It is generally believed that microbial colonization of the gastrointestinal tract (GIT) begins at birth with the maternal inoculum transferred during delivery and breastfeeding imprinting the early infant microbiota [54, 55]. Perinatal factors such as mode of delivery, diet, genetics, the environment, and geographical factors may also influence microbial composition [56]. The microbial diversity increases and converges in the first 2–5 years of life towards an adult-like microbiota, along with the development of the intestinal epithelium and the secreted intestinal mucosal barrier, which supports the mutual relationship with the commensal gut microbiota and provides a barrier against pathogenic organisms. Gut microbes benefit the host by fermenting dietary fiber into short-chain fatty acids (SCFAs), such as acetic, propionic and butyric acids, which are then absorbed by the host [57], and in synthesizing vitamin B and vitamin K as well as metabolizing bile acids, sterols, and xenobiotics [58]. Once established, the composition of the gut microbiota is relatively stable, however, lifetime events such as infections, antibiotics treatment, and long-term dietary changes can lead to microbial dysbiosis [52].

Characterization of the human intestinal microorganisms was performed conventionally by traditional culture-based methods, limiting the output range to cultivable microbes only. The advances in next-generation sequencing (NGS) technologies have enabled the intestinal microbiota genomes to be fully evaluated [59–61]. The most widely used method of microbiome research relies on the amplicon sequencing of the 16S rRNA gene [62].

### Characteristics of the gut microbiota in coeliac disease

Maintaining a diverse and well-balanced (homeostatic) gut microbial population is essential for good health. Pathogenic and symbiotic microbiota coexist in a healthy body, however, disturbance in that balance can result in dysbiosis due to various reasons. Several studies have suggested that intestinal dysbiosis drives the pathogenesis and progression of chronic GIT diseases such as inflammatory bowel disease (IBD), colorectal cancer (CRC), irritable bowel syndrome (IBS) among others [63]. Indeed, some recent studies have shown that compared to healthy subjects patients with coeliac disease have an altered composition of gut microbiota [64]. Also, although the majority of patients diagnosed with coeliac disease showed clinical improvement following strict adherence to a gluten-free diet (GFD), there is a subgroup of patients with coeliac disease who do not respond to GFD [65]. Patients with persistent symptoms of coeliac disease with long-term GFD have been shown to have an altered microbial gut composition [66] with significant differences between patients with classical gastrointestinal symptoms (such as weight loss, diarrhea, distended abdomen) and patients with extraintestinal manifestations (such as anemia, malabsorption of iron, folate, Vitamin D, calcium, short stature) [65]. Although coeliac disease in the vast majority of patients involves the proximal small intestine, it may extend distally into the ileum for a variable length [67]. It was confirmed that 66.6% of coeliac disease patients had an extension of the changes in the mucosa beyond the proximal small intestine [68]. Indeed, patients with extra-intestinal manifestations at diagnosis have a diffuse and severe grade of intestinal mucosal atrophy compared to patients with gastrointestinal symptoms only [69]. Such situations will significantly alter the composition of the gut microbiota that resides in both the small bowel and the colon.

The data characterizing the gut microbiota in coeliac disease patients was derived from both fecal samples and duodenal mucosa biopsies in studies done mostly in children with coeliac disease [64, 70–81] with fewer studies being done in adults [78, 82–85]. These studies showed differences in the microbial composition of fecal and duodenal samples in children and adults when comparing active coeliac disease with patients with treated coeliac disease, or healthy controls [86]. The most abundant bacterial phylum in adults with coeliac disease was *Firmicutes*, whereas *Proteobacteria* are present mainly in children with coeliac disease [86]. *Bacteroidetes* and *Actinobacteria* were among the other major phyla shared by both adults and children [86]. Most studies were conducted using different methods such as culture-based methods in combination with or without other technologies, denaturing gradient gel electrophoresis [DGGE], fluorescence hybridization [FISH], temporal temperature gradient gel electrophoresis [TTGE] and real-time polymerase chain reaction [RT-PCR], and consequently failed to identify all microbial taxa. The variability in the sample size of the studies and the age of the participants also makes interpretation of the findings difficult. In this review, we have summarized some of the major studies including those using culture-based methods and PCR or 16S rRNA sequencing to generate microbial signatures in children (Table 1) and adults (Table 2) using either fecal samples or duodenal biopsies or both.

**Table 1:** Microbial signatures in children with active coeliac disease relative to healthy controls^a^

Study	Sample source	Subject details	Methodology	Conclusion (microbiota signatures in coeliac disease) compared to controls
[75]	Stool	26 coeliac patients and 23 controls	Culture	^#^Bacteroides ^#^Staphylococcus ^#^Clostridium
[76]	Stool and duodenal biopsies	30 Coeliac patients, 30 controls	PCR	Stool sample: ^$^*Bifidobacterium count* ^$^*Bifidobacterium Longum* Duodenal biopsies**:** ^$^*Bifidobacterium count* ^$^*Bifidobacterium Longum* ^$^*Bifidobacterium cantenulatum* ^#^*B. lactis*
[77]	Stool and duodenal biopsies	25–30 Coeliac patients, 8–30 controls	PCR	Stool sample: ^#^*E. coli* prevalence and count ^$^*Bifidobacterium* count ^##^*Clostridium leptum* counts ^#^*Staphylococcus* prevalence and counts Duodenal biopsies: ^$^Bifidobacterium count ^#^Bacteroides counts ^#^Clostridium leptum ^#^E. coli count ^#^Staphylococcus counts ^$^*C. coccoides* prevalence
[79]	Duodenal biopsies	20 Coeliac patients, 10 controls	PCR	^#^*Bacteroides vulgatus* prevalence ^#^*E. coli* prevalence
[74]	Stool sample and duodenal biopsies	19 Coeliac patients, 15 controls	PCR/Culture	Stool sample and duodenal Biopsies: ^#^Eubacteria diversity ^$^*Lactobacillus* counts ^$^*Enterococcus* counts ^$^Bifidobacterium counts ^#^Bacteroides ^#^Porphyromonas ^#^Prevotella ^#^*Staphylococcus*
[193]	Stool sample	20 Coeliac patients, 20 controls	PCR/Culture	^#^*Staphylococcus epidermidis* ^#^*Staphylococcus haemolyticus* ^$^*Enterococcus faecium*
[89]	Duodenal biopsies	32 Coeliac patients, 8 controls	Culture/ 16S rRNA sequencing	^$^*Streptococcus mutans* ^$^*Streptococcus anginosus* ^$^*Firmicutes* ^#^*Staphylococcacea* ^#^*Staphylococcus epidermidis* ^#^*Staphylococcus pasteuri* ^$^*Streptococcaceae* ^#^*Proteobacteria* ^#^*Enterobacteriaceae* ^#^*Klebsiella oxytoca*
[64]	Duodenal biopsies	10 Coeliac patients, 9 controls	16S rRNA sequencing	^#^*Prevotella melaninogenica* ^#^*Haemphilus* ^#^*Serratia* ^$^*Prevotella oralis* ^$^*Proteus* ^$^*Clostridium stercorarium* ^$^*Ruminococcus bromii* ^$^*Papllibacter cinnamivorans*
[78]	Duodenal biopsies	8 Coeliac Disease, 5 controls	16S rRNA gene sequencing	^$^Streptococcus ^$^Prevotella ^#^Neisseria ^#^Haemophilus

^a^This table only includes the comparison of the patients with active or untreated Coeliac disease and healthy controls

^#^Increase in relative abundance

^$^Decrease in relative abundance

**Table 2:** Microbial signatures in adults with active coeliac disease relative to healthy controls^a^

Study	Sample source	Subject details	Methodology	Conclusion (microbiota signatures in coeliac disease) compared to controls
[78]	Stool sample	10 Coeliac patients, 11 controls	PCR	^$^*Lactobacillus* diversity ^$^*Lactobacillus sakei* ^$^*Bifidobacterium*
[84]	Duodenal biopsy*	10 Treated Coeliac patients with persistent symptoms, 10 treated Coeliac patients’ symptoms free	16 S rRNA sequencing	^#^Proteobacteria ^$^Bacteroidetes ^$^Firmicutes
[85]	Duodenal biopsy	6 Coeliac patients, 11 controls	16 S rRNA sequencing	^$^Bacteroidetes ^$^Fusobacteria
[82]	Stool sample and Duodenal biopsy	23 Coeliac patients, 24 controls	16S rRNA gene sequencing	Stool sample: ^$^Akkermanisia ^$^Dorea Duodenal biopsy: ^#^Helicobacter ^#^Megasphaera
[78]	Duodenal biopsy	5 Coeliac Disease, 5 controls	16S rRNA gene sequencing	^#^*Mycobacterium spp* ^#^*Methylobacterium spp*

This table only includes the comparison of the microbial profiles in patients with active or untreated Coeliac disease and healthy controls. However, since very few studies are available in adults, we included one study^**a**^ that allowed us to compare the treated coeliac patients (with persistent symptoms) with symptoms free treated Coeliac patients

^#^Increase in relative abundance

^$^Decrease in relative abundance

One of the initial studies aimed at identifying specific bacterial groups responsible for the gut microbiota alterations in children with the active coeliac disease found that rod-shaped bacteria were frequently found in small bowel biopsies of children with active and inactive coeliac disease, compared to controls [87]. Using culture-based methods, Collado et al. concluded that the fecal samples collected from children with coeliac disease had significantly higher levels of genera *Bacteroides*, *Clostridium*, and *Staphylococcus* when compared to healthy subjects [75]. Collado et al. followed up their results with PCR—based analysis, using both fecal samples and duodenal biopsies collected from pediatric patients with active and non-active coeliac disease and healthy controls [76, 77]. They showed that the intestinal microbiota in untreated children with coeliac disease is unbalanced and was partially restored after long-term adherence to GFD [76, 77]. This imbalance was observed in both feces and intestinal biopsies in terms of reduction in the abundance of total *Bifidobacterium* count and species-specific *Bifidobacterium longum* [76, 77]. A Swedish study further explored the coeliac disease-related dysbiosis and found higher numbers of rod-shaped bacteria (*Clostridium *spp., *Prevotella *spp., and *Actinomyces *spp.) in the small bowel mucosa of coeliac disease patients [88]. Other studies have reported a higher abundance of the *Proteobacteria* phylum (family *Enterobacteriaceae*), genera *Bacteroides* and *Staphylococcus* in the untreated coeliac disease when compared to healthy subjects [77, 80, 89]. Many studies used fecal samples in addition to the duodenal samples, often showing an imbalance between gram-negative bacteria and gram-positive bacteria (G-/G +) ratio marked by an increase in *Bacteroides* and *Proteobacteria*, and a decrease in *Lactobacillus* and *Bifidobacteria* in feces of patients with coeliac disease compared with controls [77, 90–93].

The genus of *Bacteroides* is a major component of the human intestinal microbiota and is generally considered symbiont, although some species have been involved in disrupting the integrity of the intestinal epithelial barrier and thus exhibiting pro-inflammatory effects [94–96]. Multiple studies in children and adults have reported an increase in the relative amount of gram-negative *Bacteroides*, *Prevotella* *species*, *Escherichia. coli* [64, 74, 77, 79, 84] and low levels of the protective anti-inflammatory bacteria such as *Bifidobacteria* and *Lactobacilli* [78, 83, 84, 97]. This suggests that gut microbiota could affect the pathogenesis and coeliac disease progression by activating the innate immune system, modulating the function of the epithelial barrier, and driving mucosal inflammation as discussed in more detail in the next section. Although the above studies have provided us with a general makeup of coeliac disease microbiota signatures and suggested a coeliac disease-driven dysbiosis, there is still a long way to go in answering specific questions such as whether these microbial changes precede the disease, are associated with the onset of gluten-driven disease or are the result of inflammation or other related immune cell phenotypes.

Caminero et al. using mice populated with the human intestinal bacteria showed that the microbe-gluten-host interaction may modulate the autoimmune risk in genetically susceptible subjects and that the microbiome of coeliac disease patients processes gluten differently compared to the microbiome of healthy subjects [98]. Associations have also been established between the HLA-DQ2/DQ8 genotype and the gut microbiota composition, by profiling the gut microbiota of genetically at-risk children [99]. It has been shown that high-risk children carry specific microbiota compared with children at low genetic risk for coeliac disease [100], suggesting that the microbiota may also serve as a predisposing factor for coeliac disease. Several studies have highlighted the central role of gluten intolerance and microbial dysbiosis in patients with coeliac disease [7, 76, 86]. Changes in the abundance of various *Firmicutes* and *Bacteroidetes* have been reported in patients with active coeliac disease. Also, the proportion of gluten-proteolytic bacteria such as *Bifidobacterium, Lactobacillus, Rothia spp* is decreased [76, 101, 102], while the abundance of Gram-negative *Proteobacteria* (*E. coli* and *Enterobacteriaceae*) is increased [18]. This results in altered exposure of intestinal epithelial to bacterial lipopolysaccharides (LPS) and metabolites such as SCFAs [18]. The dysbiotic microbiota may result in increased amounts of bacterial LPS in the intestine resulting in the dysregulated immune response that is evident through the activation of various intraepithelial lymphocytes (IELs) subsets and epithelial cells that act as a trigger for increased production of anti-microbial peptides (AMPs) and mucin [103, 104]. Furthermore, altered microbial metabolites can induce T_Reg_ cells and dendritic cells [105–108] which in turn produce IL-10 and retinoic acid which activates various cellular functions within the lamina propria (Fig. 2).

The above data suggest a multifactorial etiology of coeliac disease in which the gut microbiota appears to be a critical player, with the overall consensus suggesting a coeliac disease association with over-representation of pathobionts and a decrease in protective symbionts and/or commensals numbers. However, further complicating this scenario is the complexity of the microbiota and its dependence on environmental and individual genetic factors. It is difficult to conclude, based on current evidence, whether changes in the composition and function of the microbial community in patients with coeliac disease are the cause or effect of the disease. Most studies of gut microbiota profiling use fecal samples as a proxy because of the noninvasiveness and the convenience of stool sampling, and the same seems to be the case with many studies of coeliac disease. However, it is important to remember that fecal samples are unlikely to represent the actual microbial composition which resides in the duodenum or along the surface of the duodenal mucosa. Therefore, it is advisable to analyze tissue biopsies in addition to fecal samples to build a comprehensive picture of the microbial diversity in coeliac disease research. Furthermore, prospective, multicenter, longitudinal studies mixing basic and applied research using “omics” approaches are required to explain the role of the gut microbiota as an additional factor involved in the onset of autoimmune diseases such as coeliac disease.

### Virome in coeliac disease

The involvement of bacterial dysbiosis in modulating the intestinal homeostasis has been well described in various human diseases, however, the role of the virome, which is also an integral part of the human microbiota, is often overlooked. The human virome comprises a diverse collection of endogenous retroviruses, eukaryotic viruses, and bacteriophages [109] and is progressively recognized as orchestrater of the intestinal barrier integrity and gut homeostasis. Changes in the enteric virome composition and their role in modulating the intestinal microbial diversity and functionality have been emphasized in several recent studies including in coeliac disease [110–114]. Many common viral infections play a role in disrupting the intestinal functions of the host and thereby causing various human diseases. Yet their role in causing coeliac disease is still debatable. The potential role of early viral infections, in particular adenovirus, enterovirus, rotavirus, and reovirus, in coeliac disease pathogenesis has been suggested primarily based on cross-sectional or epidemiological studies [115–118]. However, the evidence remains inconclusive due to the retrospective nature of the study, reverse casuality, and in some cases inconsistensy of later studies [119, 120]. Bouziat et al. observed a trend in higher levels of anti-reovirus antibodies in patients with coeliac disease, suggesting a link between reovirus infection and pathogenesis of coeliac disease [121]. In this study, reovirus-infected mice developed a protective immunity to reovirus post-infection, though they suffered a loss of tolerance to dietary gluten [121]. Two prospective longitudinal birth cohort studies have recently pointed in the same direction; the first concluded that a higher prevalence of enterovirus, but not adenovirus, was associated with a later onset of coeliac disease during early childhood [122]. A nested case–control cohort from the TEDDY study showed that the cumulative number of enteroviral stool exposures between 1 and 2 years of age along with a higher gluten intake was associated with coeliac disease autoimmunity [123]. These results indicate that in genetically predisposed children, enteroviral exposure increased by a higher intake of gluten could act as triggers of coeliac disease. Collectively, these results proposed a mechanism by which viral infections early in life, when the gut microbiota evolves, may probably lead to an altered microbial state, affecting the intestinal immune responses. These in turn disrupt the mucosal barrier resulting in increased translocation of gluten peptides into the mucosa leading to the loss of immune tolerance. Experimental studies are required to confirm the validity of enterovirus as a trigger factor and also the option of vaccination in reducing the risk of development of coeliac disease.

### Oral and blood microbiome in Coeliac disease

Despite the role of oral microbes in gluten degradation [124], coeliac microbiome research has focused mostly on the intestinal microbiota. The gastrointestinal system begins with the oral cavity and leads to the esophagus and extends to the anus via the stomach, small and large intestines. The oral cavity is one of the most diverse body sites harboring more than 700 different bacterial species that are quite distinct from the gut microbiota [125], daily salivary secretions and subsequent swallowing carry 8 × 10^10^ bacteria (that shed from the oral mucosal surfaces per day) to the GI tract [126]. Thus, the salivary, duodenal, and fecal microbial analysis could capture comprehensive disease-specific microbial changes along the entire gastrointestinal tract that could be highly useful for understanding food-sensitive enteropathy diseases such as coeliac disease.

Gluten, consisting of glutenins and gliadins, contains structurally relatively high concentrations of glutamine and proline residues [127], rendering it highly resistant to degradation by human gastrointestinal proteases. Examples of these protease-resistant peptides of particular concern are a 33-mer derived from α-gliadins and a 26-mer derived from γ-gliadins [128] that apart from being resistant to proteolysis, contain multiple copies of the highly immunogenic epitopes which stimulate CD4^+^ T cells in the lamina propria of HLA-DQ2-positive patients with coeliac disease [129]. A mixture of naturally occurring bacteria in the human dental plaque may, however, cleave gliadins, including the immunogenic 33-mer and 26-mer domains [130]. Few studies that have analyzed coeliac disease-related oral microbiome, reported that the salivary microbiota and metabolome are associated with coeliac disease [131]. Oral microbiota showed an increased number of salivary *Lactobacillus* sp. in patients with active and refractory coeliac disease compared with healthy controls. This suggests its possible role in increased salivary gluten-degrading activity in patients with coeliac disease [132]. Comparison of oropharyngeal samples from coeliac disease patients and control subjects showed a higher abundance of phyla *Proteobacteria* at the cost of *Bacteroidetes* in coeliac disease patients than in control subjects [133]. The above studies pointed towards a possible relationship between gluten and the coeliac disease-associated dysbiosis, in the oral cavity with a continuum to the intestinal mucosa, but this needs to be clarified with further investigation.

Though debatable, the concept of a blood microbiome emerges with cumulative evidence [134, 135]. Blood has long been considered a sterile environment with no proliferative microbes [136], but it has now been shown to harbor numerous species of dormant bacteria that are not readily culturable and that may constitute a healthy human blood microbiome (HBM) [137]. It has been suggested that aberrant blood microbiota plays a role in various diseases such as type II diabetes and cardiovascular disease [138, 139]. The concept of “Atopobiosis” was put forward to describe the migration of the gut or oral microbes to the bloodstream which can lead to the dynamics of many inflammatory diseases [135]. In a recent study, the alteration in blood microbiome composition and taxonomic diversity was observed in adult coeliac disease patients compared with healthy subjects [140]. Damaged, inflamed intestines with increased permeability are typical of patients with coeliac disease, so is the likelihood of developing a unique blood microbiome that could indicate intestinal damage and influence the response to gluten. The above hypothesis remains speculative and further studies involving larger cohorts are required to better define the role of the blood microbiome as a biomarker for patient classification, diagnosis and for therapeutic stratifications.

## Environmental factors in coeliac disease

### Birth mode

The human GIT is continuously exposed to numerous external stimuli, including food antigens, pathogenic, and commensal microbes, providing the largest interfaces in the human body for host–pathogen interactions. According to the widely accepted hypothesis, the incoherent nature of external factors, including eating habit, diet, exposure to broad-spectrum antibiotics or other toxic chemicals can lead to a breakdown in the intestinal homeostasis by influencing the composition of the gut microbiota as well as the epithelial immune and barrier functions [141–143]. The birth mode is considered the critical determinant of early colonization of the neonatal gut microbiome [144]. The Cesarean section commonly thought to have a permanent impact on the intestinal microbiota of infants, due to the lack of exposure to the maternal vaginal and gut microbiota [145]. Studies have indicated that there is an association between cesarean section and an increased risk of coeliac disease [146–148]. Differences in elective cesarean section where babies are not exposed to the maternal microbiota have been observed [149]. In the case of an emergency Cesarean section, the risk of amniotic sac rupture exists, which exposes the baby to the vaginal microbes, such alterations in the intestinal microbiota during the first year of life can result in immune dysregulation and increase the risk of coeliac disease. However, other studies have found inconsistent conclusions and limited or no evidence to support any association between the birth mode and coeliac disease [147, 150]. We have summarized the finding of the major studies in Table 3.

**Table 3:** Overview of studies included in this review that have linked birth mode with the development of coeliac disease

Study	Type of study	Subjects	Main findings
[146]	Case control from Germany	157 coeliac disease cases, 862 controls	Children with coeliac disease had significantly high likelihood of being born by cesarean delivery compared with control subjects (odds ratio: 1.8 [95% confidence interval 1.13–2.88]; *P* = 0.014)
[147]	Prospective birth cohort study from Norway	650 children with coeliac disease and 107,828 controls	Coeliac disease was not associated with mode of delivery (cesarean section, model 1: OR, 0.84; 95% confidence interval [CI] 0.65–1.09, and model 2: OR, 0.83; 95% CI 0.63–1.09)
[148]	Birth Registry-based study from Sweden	6596 children of who developed coeliac disease before 15 years of age	Among boys, elective caesarean delivery increased the risk of coeliac disease (OR 1.2; 95% CI 1.0–1.4)
[149]	Population-based birth cohort study from Sweden	11,749 coeliac disease and 53,887 age- and sex-matched controls	Positive association between elective cesarean delivery and later coeliac disease (adjusted odds ratio [OR], 1.15; 95% confidence interval [CI] 1.04–1.26)
[194]	Birth Registry-based study from UK	90 children with coeliac disease and 24,8431 children for whom there was no record of admission for coeliac disease	No significant association between coeliac disease and cesarean birth (odds ratio = 0.29; 95% CI 0.07–1.17) (*P* = 0.064)
[195]	Birth Registry-based study from Denmark	1944 children with coeliac disease	No significant association between coeliac disease and cesarean birth (PARF 0.99 (0.87–1.14); (*P* = .89)
[196]	Birth Registry-based study from Sweden	3817 children with coeliac disease 191 with had both coeliac disease and T1DM	The increased risk of having a double diagnosis of type 1 diabetes and coeliac disease was associated with being born by Caesarean sections (odds ratio 1.60 (1.07–2.39)
[150]	population-based birth cohort from Italy	1,227 children with coeliac disease, 220230 controls	No significant association between coeliac disease and planned or un-planned cesarean birth
[197]	Multinational birth cohort:The TEDDY study	979 with CDA and 343 with coeliac disease	C-section is not associated with increased risk coeliac disease (HR = 0.85; 95% CI 0.65, 1.11; *P* = 0.24) and coeliac disease autoimmunity (CDA) (HR = 0.91; 95% CI 0.78, 1.06; *P* = 0.20)
[198]	Registry-based study from Denmark and Norway	Denmark: (n = 1,049,633) Cesarean sections (*n* = 196,512) coeliac disease (*n* = 1,395) children Norway: (n = 537,457) Cesarean section (*n* = 90,128) coeliac disease (*n* = 1,919)	Mode of delivery was not associated with an increased risk of diagnosed coeliac disease. Odds ratio1.11 (95% CI 0.96–1.29) in the Danish cohort and 0.96 (95% CI 0.84–1.09) in the Norwegian cohort

### Use of antibiotic

Several studies have shown that exposure to antibiotics can have a significant impact on the composition and functionality of the human gut microbiota [151]. Antibiotics can modulate the gut microbial composition and influence the development of chronic diseases [152]. Children exposed to antibiotics displayed a delayed maturation of microbiota compared with those not exposed to antibiotics [153].

Such alterations in the developing gut microbiota influence the immune maturation process and predispose to coeliac disease [15]. Several studies have pointed towards a link between early intestinal infection and antibiotic usage with intestinal dysbiosis, alteration in the population of sub-lymphocytes [154], and coeliac disease onset [150]. Recent observational studies that found that subjects treated with antibiotics are more likely to develop coeliac disease later in life, further support this theory. In a nationwide registry-based cohort study, Sander and colleagues collected data on more than 1.7 million children born in Denmark and Norway including 3346 diagnosed with coeliac disease. They found that exposure to antibiotics in the first year of life was positively associated with a later diagnosis of coeliac disease [155]. Interestingly, a dose-dependent association was also found between the increasing number of antibiotics used and the risk of coeliac disease [155]. Antibiotic use has also been shown to be significantly associated with the development of coeliac disease in Swedish [156] Italian [150], and North American children [157]. The dysbiosis induced by antibiotics was characterized by a decrease in the *Bifidobacteria longum* counts and an increase in the counts of *Bacteroides fragilis* [154]. Several previous studies have identified the decrease in *Bifidobacterium* as a characteristic of dysbiosis in patients with coeliac disease [83, 90, 91]. Collectively, these data suggest that dysbiosis resulting from antibiotics use can influence the development of coeliac disease. An overview of the studies that have linked the use of the antibiotics with the development of coeliac disease is shown in (Table 4.)

**Table 4:** Overview of studies included in this review that have linked antibiotic use with the development of coeliac disease

Study	Type of study	Subjects	Main findings
[157]	Case control from USA	332 cases, 241 controls	Early life exposure to antibiotics associated with coeliac disease (adjusted or 1.133, 95% CI 1.032–1.244; *P* = 0.007)
[155]	Register-based cohort study from Denmark and Norway	1.7 million	Exposure to systemic antibiotics in the first year of life was associated with coeliac disease (pooled OR 1.26, 95% confidence interval 1.16–1.36)
[156]	Nationwide case–control study from Sweden	2933 cases, 14,571 controls	Antibiotic use was associated with coeliac disease (OR = 1.40; 95% CI [1.27–1.53),
[150]	Population-based birth cohort study from Italy	203,000 babies	Antibiotic use was significantly associated with coeliac disease onset (incidence rate ratio IRR = 1.24, 95% CI 1.07, 1.43)
[117]	Population-based incident case-referent study	(*n* = 97) treated with antibiotic during the first 6 months of life. (*n* = 134) untreated	No significantly increased risk for coeliac disease was seen regarding antibiotic treatment (OR 1.2; 95% CI 0.87–1.6; *P* = 0.27)
[118]	Multinational prospective birth cohort	8495 children	Cumulative use of any antibiotic during the first 4 years of life was not associated with the appearance of any coeliac disease autoantibody (hazard ratio [HR], 0.98; 95% CI 0.95–1.01) or the transglutaminase autoantibody (HR, 1.00; 95% CI 0.98–1.02)

### Diet

“Let food be thy medicine” is the concept that was first proposed by Hippocrates in 400 BC, to emphasize the importance of dietary nutrients in preventing or curing diseases. Therapeutic utility of diet, dietary modulation experiments have become a method of choice for both understanding disease etiology [158] and devising personalized treatment strategies [159–161]. Studies have highlighted the importance of dietary interventions in modulating the gut microbiota and eliciting specific effects [162]. As discussed in the previous sections, the intestinal microbiota is exposed to a highly dynamic environment that can be influenced by different co-variants [141–143]. For example, the microbiota is routinely challenged by a complex mix of millions of dietary compounds and common medicinal products [163]. Antibiotics and even non-antibiotic medicines such as metformin are widely recognized to have a side effect on the microbiota composition [164–166]. Similarly, several dietary components, such as vitamins, hormones, and other micronutrients, have been reported to impact the gut microbiota, which may influence the intestinal homeostasis and host health [52, 167]. Of the multiple exogenous and endogenous host co-variant factors, diet and its contents appear to be the most dominant factors in affecting the microbial composition. Sudden and abrupt dietary changes, such as switching to animal-based or plant-based diets, have been shown to modify the microbial composition in just 24 h of experimental initiation, but the discontinuation of such diet resulted in the reversion to baseline within 48 h [162, 168]. Among the common bacteria that are affected by diet, are *Bifidobacterium* *spp*., *Lactobacillus* *spp*.*, Bacteroides spp*., *Clostridium spp*., *Eubacterium spp*., *Enterococcus* *spp*., *Escherichia coli, and Streptococcus* *spp*. [162, 169]. Many of these microbes are associated with coeliac disease pathology as discussed. Considering the profound contribution of diet in the regulation of microbiota and its crucial role in the orchestration of human health, different health organizations across the world have set guidelines for a “healthy diet” that specifies the defined intake of micro and macronutrients and addresses common diseases such as IBD, IBS, and coeliac disease, although such recommendations are often conflicting [170]. GFD, the key therapy for coeliac disease, has been shown to only partially restore the unbalanced gut microbiota of coeliac disease patients even after long-term treatment [77, 91]. This may be because a GFD is not always completely gluten-free. There is a debate over what a GFD should be, but a diet of up to 20 parts per million gluten is usually taken into account [171]. Strict adherence to the GFD in coeliac patients has been reported to heal the damaged intestine rapidly and improve the symptoms of the disease [172]. However, patient compliance with the therapy is highly variable, as many patients face difficulties following GFD, and eating gluten again may trigger disease relapse [173] and in some patients, GFD was found to worsen the overall microbiota diversity [70]. De Palma et al. studied the effects of a GFD on the composition of the gut microbiota in ten healthy subjects where they found that GFD was associated with a reduction of beneficial gut bacteria [174]. The possible reason for this may be the fact that gluten exerts a prebiotic action [175]. Therefore, the development of novel therapeutic approaches such as supplementation of GFD with pre- or probiotics is a promising approach to improve the clinical management of coeliac disease patients under GFD.

## Microbiota as a potential therapeutic target for coeliac disease

The aim of therapeutic modulation of the gut microbiota composition is to establish and maintain eubiosis. There are various methods which can be usually used. Those include dietary regulation, prebiotics, probiotics, and faecal microbial transplantation [163, 176, 177].

### Probiotics and prebiotics as strategies to modulate microbiota in coeliac disease

Recent studies have suggested that a diet with a low content of fermentable oligosaccharides, disaccharides, monosaccharides, and polyols (FODMAPs) could be beneficial for patients with coeliac disease. In a small randomized controlled trial, Roncoroni et al. showed that a low FODMAP GFD improves the psychological health and gastrointestinal symptoms of adults with coeliac disease after 21 days compared to standard GFD [178]. Whilst the long-term effect of such a diet in coeliac disease is not clear, low FODMAP diets have recently been reported to be associated with alterations in the gut microbiota [179, 180].

The fact that microbial imbalances persist despite of a GFD has led to the consideration of other therapeutic approaches, such as the introduction of probiotics to the GFD. Probiotics are live microorganisms that when consumed in sufficient quantities confer health benefits. There are several reasons why probiotics might be useful in coeliac disease. Certain probiotic strains can safely activate an immune response, without inducing inflammation [181]. *Lactobacilli-*made Bacteriocins result in the formation of pores in the plasma membrane of the pathogenic bacteria and promoting cell lysis [181]. Probiotics also mediate increased expression of occlusion zone proteins, allowing the proper functioning of the tight junctions [181]. In an animal model of coeliac disease, the administration of *Bifidobacteria* led to the downregulation of mRNA expression of pro-inflammatory markers and reduced production of NF-kB, TNF-alpha, and IL-1beta [182]. Lindfors et al. studied the effects of two probiotic strains (*Lactobacillus fermentum* and *Bifidobacterium lactis*) on gliadin toxicity in intestinal epithelial cells [183]. They found that *B. lactis* reduces intestinal permeability induced by gliadin, while *L. fermentum* did not affect this parameter. The authors emphasized the usefulness of this probiotic in coeliac disease patients who do not respond to the GFD [183].

Nonetheless, there have been contradictory reports from clinical studies examining the efficacy of probiotics in patients with coeliac disease. Data from a small randomized trial showed that a strain of *Bifidobacterium infantis* improved gastrointestinal symptoms in adults with the untreated coeliac disease such as indigestion, bowel constipation, and reflux but did not improve intestinal permeability [184]. In another trial, Olivares et al. showed that a strain of *Bifidobacterium longum* improved the height percentile of children with coeliac disease but did not improve the gastrointestinal symptoms [185]. We have summarized the human studies that have examined the role of probiotics in coeliac patients in (Table 5). The inconsistencies in the findings of these studies may be due to the differences in the cohort selection and size of the sample, study length, type, and dosage of probiotics used. In addition to the direct administration of probiotics to patients with coeliac disease, some researchers have also examined the use of microorganisms as components of the food products. Di Cagno et al. reported that patients with coeliac disease who consumed Sourdough wheat bread containing probiotic strains *L. alimentarius*, *L. brevis*, *L. sanfranciscensis*, and *L. hilgardii*, showed reduced symptoms with improved gluten tolerance [186]. Similar studies done with the pretreatment of wheat flour with probiotic bacterial strains have shown promising results [187, 188].

**Table 5:** Overview of studies that have investigated diet-based Probiotic and or Prebiotic interventions in the treatment of coeliac disease in Humans

Study	Type of study	Subjects	Main findings
[199]	Prospective, double- blind, randomized placebo-controlled parallel group study from Italy	109 coeliac disease adult patients with IBS-type symptoms on strict Gluten free diet. (*n* = 54) received the probiotic blend of five strains and (*n* = 55) in the placebo group	A 6-week probiotic treatment is effective inproved severity of IBS-type symptoms, in coeliac disease patients on strict GFD, also resulted in the modification of gut microbiota, specifically increase of B*ifidobacteria*
[200]	Cohort based study from Brazil	14 coeliac patients and 17 healthy subjects with both groups on daily intake of 100gms of probiotic-containing yogurt for 30-day period	Probiotic yogurt intake lead to a significant increased the Bifidobacteria number in coeliac disease patients, but not in healthy participants
[201]	Cohort based study from Argentina	41 adult participants (*n* = 24), coeliac disease active, no treatment; (*n* = 12), coeliac disease active with *Bifidobacterium infantis* Natren Life Start super strain (NLS-SS) for 6 weeks (*n* = 5), coeliac disease 1 year on GFD	*B. infantis* treatment decreased the innate immune markers, macrophage counts, Paneth cell counts and α-defensin-5 in coeliac disease patients. However, GFD was more effective than *B. infantis* in decreasing the duodenal macrophage counts in coeliac disease patients
[184]	Placebo controlled, double blind study from Argentina	22 adults with untreated coeliac disease; (*n* = 12) received the probiotic capsule *Bifido bacterium* natren life start (NLS), (*n* = 10) placebo for 3 weeks	*B. infantis* is safe to use and may alleviate some symptoms in untreated coeliac disease. Overall probiotic treatment resulted in some immunologic changes but did not modify abnormal intestinal permeability
[202]	Randomized control trail from Australia	45 adult patients with coeliac disease, Participants took 5 g of VSL#™ probiotic formulation (*n* = 23) or 5 g placebo (*n* = 22) orally twice daily for 12 weeks	The probiotic formula when taken orally over the 12-week period did not significantly alter the microbiota measured in this population
[187]	Randomized trail from Italy	13 patients with coeliac disease (*n* = 6) assigned to natural flour baked goods (NFBG),(*n* = 7) assigned to hydrolyzed flour baked goods with sourdough lactobacilli for 60 days	Hydrolyzed wheat flour, manufactured with sourdough lactobacilli and fungal proteases, was safe for consumption and was not toxic to patients with coeliac disease
[203]	A prospective cohort study with from USA, Finland, Germany and Sweden	6520 genetically susceptible children with 1460 reporting the probiotic use in the first year of life	Overall exposure of probiotics during the first year of life was not associated with CDA or coeliac disease. However, intake of probiotics via dietary supplements was associated with increased risk of coeliac disease Autoimmunity (CDA)
[189]	Randomized, Placebo-Controlled Trial from Poland	34 paediatric coeliac disease patients on GFD. (*n* = 18) receving prebiotic (Synergy 1) or placebo (maltodextrin) daily for 3 months	*Bifidobacterium* count increased significantly (*P* < 0.05) in the Synergy 1 group. Along with an increase in faecal acetate and butyrate levels was observed in the prebiotic group
[204]	A double-blind placebo-controlled study from Slovenia	40 coeliac disease children and 16 healthy children. Group of 20 each coeliac disease children received probiotic formulation (a mixture of 2 strains, *B. breve* BR03 (DSM 16,604) and *B. breve* B632 (DSM 24,706) in ratio 1:1)and the other placebo for 3 months	Probiotic administration had a negative correlation between *Verrucomicrobia*, some unknown phyla of Bacteria, Synergistetes, Euryarchaeota and some SCFAs, identifying them as potential target in microbiome restoration process
[205]	Double-blinded, placebo-controlled study from Slovenia	40 coeliac disease children on GFD before and after probiotic (*Bifidbacterium breve strains B632 and BRO3)* or placebo administration and 16 healthy children (Control group) for 3 months	Probiotic resulted in an increase of *Actinobacteria along with the Firmicutes/Bacteroidetes* ratio. Concluding that 3-month administration of *B. breve* strains helps in restoring the healthy percentage of major microbial components
[206]	Double blind placebo-controlled trial from Slovenia	49 coeliac disease children on gluten-free diet (GFD) and 18 healthy control. (*n* = 24 children with coeliac disease) daily received *B. breve* BR03 and B632) and the second group (*n* = 25 children with coeliac disease) received placebo for 3 months	Probiotic intervention with *B. breve* strain resulted in a decrease the production of pro-inflammatory cytokine TNF-α in children with coeliac disease on GFD
[185]	Double blind, randomized, placebo-controlled trial from Spain	33 coeliac disease children on GFD received a capsule containing either *B. longum* CECT 7347 (10^9^ colony-forming units) or placebo (excipients) daily for 3 months	Probiotic treatment showed greater height percentile, decreased peripheral CD3^+^ T lymphocytes, and slightly reduced TNF-α concentration. The number of *Bacteroides fragilis* and content of secretory IgA in the stool was also reduced by the probiotic treatment. The small sample size is a limitation of the study

To promote the growth of ‘beneficial’ bacteria, specific prebiotics have been added to the GFD and were evaluated in clinical trials. A recent study found that when added to the GFD, oligofructose-enriched inulin (Synergy 1), a prebiotic, increased *Bifidobacterium* counts, and the levels of acetate and butyrate in the gut, with no side effects [189]. Finally, the above studies have demonstrated the safety of using pre and probiotic in coeliac disease patients, additional studies are required to confirm the efficacy of probiotics and/or prebiotics as a potential adjunct in the treatment coeliac disease.

### Fecal microbiota transplantation for coeliac disease

Another approach that has been considered is the use of fecal microbiota transplantation (FMT). FMT is a mechanism by which feces from a healthy donor are infused into a recipient’s GI tract to treat a particluar disease associated with gut microbiota alteration [190]. FMT is an effective treatment for *Clostridium difficle* infections [191]. Formal studies on the use of FMT in coeliac disease are lacking, but Beurden et al. recently reported a 68-year-old with refractory coeliac disease who received FMT as a treatment for Clostridium difficile infection [192]. Interestingly, the patient’s symptoms also resolved following FMT and duodenal biopsies obtained 6 months after FMT showed complete recovery of villous atrophy.

## Conclusions

Although genetic factors such as the presence of HLA-DQ2 or HLA DQ-8, and dietary gluten exposure are necessary for coeliac disease development, they are not sufficient. Several studies have demonstrated that alterations in the gut microbial composition, such as reduced *Bifidobacteria* and *Lactobacilli* levels and increased levels of *Proteobacteria* can contribute towards the pathogenesis of the disease. However, these studies were unable to determine the cause vs consequence of the altered microbiome in Coeliac disease, as well as the mechanistic analysis on how different bacterial strains may influence the intestinal health. Multi-omics data from ongoing longitudinal cohort studies such as CDGEMM (Coeliac Disease, Genomic, Environmental, Microbiome, and Metabolomic Study) are expected to clarify some of the above questions. The role of fungi and viruses in coeliac disease pathogenesis has received little attention and warrants further investigations. The dynamic interplay between genetic, microbial, and environmental factors such as infant feeding practices, diet, antibiotics, and infections, that contribute to the development of coeliac disease will be a major focus of future research. To develop precise epidemiological modeling and clinical path, prospective studies on prevalence, risk group screening, disease classification, intervention time point analysis are needed. GFD, the key treatment for coeliac disease has been shown to only partially restore the unbalanced gut microbiota in coeliac patients even after long-term treatment while GFD itself may contribute to dysbiosis. The efficacy of potential gut microbiota modulators such as probiotics, prebiotics, and FMT as adjunctive therapies in coeliac disease is largely unproven and requires further investigation.

## References

[1] De Re V, Magris R, Cannizzaro R (2017). New insights into the pathogenesis of celiac disease. Front Med (Lausanne).

[2] Singh P, Arora A, Strand TA, Leffler DA, Catassi C, Green PH, Kelly CP, Ahuja V, Makharia GK (2018). Global prevalence of celiac disease: systematic review and meta-analysis. Clin Gastroenterol Hepatol.

[3] Lebwohl B, Sanders DS, Green PHR (2018). Coeliac disease. Lancet.

[4] Caminero A, Meisel M, Jabri B, Verdu EF (2019). Mechanisms by which gut microorganisms influence food sensitivities. Nat Rev Gastroenterol Hepatol.

[5] Ludvigsson JF, Leffler DA, Bai JC, Biagi F, Fasano A, Green PH, Hadjivassiliou M, Kaukinen K, Kelly CP, Leonard JN, Lundin KE, Murray JA, Sanders DS, Walker MM, Zingone F, Ciacci C (2013). The Oslo definitions for coeliac disease and related terms. Gut.

[6] Dubois PC, van Heel DA (2008). Translational mini-review series on the immunogenetics of gut disease: immunogenetics of coeliac disease. Clin Exp Immunol.

[7] Marasco G, Di Biase AR, Schiumerini R, Eusebi LH, Iughetti L, Ravaioli F, Scaioli E, Colecchia A, Festi D (2016). Gut microbiota and celiac disease. Dig Dis Sci.

[8] Scher JU (2016). The microbiome in celiac disease: beyond diet-genetic interactions. Cleve Clin J Med.

[9] Lionetti E, Castellaneta S, Francavilla R, Pulvirenti A, Tonutti E, Amarri S, Barbato M, Barbera C, Barera G, Bellantoni A, Castellano E, Guariso G, Limongelli MG, Pellegrino S, Polloni C, Ughi C, Zuin G, Fasano A, Catassi C (2014). Introduction of gluten, HLA status, and the risk of Celiac disease in children. N Engl J Med.

[10] Valdes AM, Walter J, Segal E, Spector TD (2018). Role of the gut microbiota in nutrition and health. BMJ.

[11] Flint HJ, Scott KP, Louis P, Duncan SH (2012). The role of the gut microbiota in nutrition and health. Nat Rev Gastroenterol Hepatol.

[12] Thursby E, Juge N (2017). Introduction to the human gut microbiota. Biochem J.

[13] Franzosa EA, Sirota-Madi A, Avila-Pacheco J, Fornelos N, Haiser HJ, Reinker S, Vatanen T, Hall AB, Mallick H, McIver LJ, Sauk JS, Wilson RG, Stevens BW, Scott JM, Pierce K, Deik AA, Bullock K, Imhann F, Porter JA, Zhernakova A, Fu J, Weersma RK, Wijmenga C, Clish CB, Vlamakis H, Huttenhower C, Xavier RJ (2019). Gut microbiome structure and metabolic activity in inflammatory bowel disease. Nat Microbiol.

[14] Han H, Li Y, Fang J, Liu G, Yin J, Li T, Yin Y (2018). Gut microbiota and type 1 diabetes. Int J Mol Sci.

[15] Olivares M, Walker AW, Capilla A, Benitez-Paez A, Palau F, Parkhill J, Castillejo G, Sanz Y (2018). Gut microbiota trajectory in early life may predict development of celiac disease. Microbiome.

[16] Chibbar R, Dieleman LA (2019). The gut microbiota in celiac disease and probiotics. Nutrients.

[17] Krishnareddy S (2019). The microbiome in celiac disease. Gastroenterol Clin North Am.

[18] Olivares M, Benitez-Paez A, de Palma G, Capilla A, Nova E, Castillejo G, Varea V, Marcos A, Garrote JA, Polanco I, Donat E, Ribes-Koninckx C, Calvo C, Ortigosa L, Palau F, Sanz Y (2018). Increased prevalence of pathogenic bacteria in the gut microbiota of infants at risk of developing celiac disease: The PROFICEL study. Gut Microbes.

[19] Sollid LM (2000). Molecular basis of celiac disease. Annu Rev Immunol.

[20] Kagnoff MF (2007). Celiac disease: pathogenesis of a model immunogenetic disease. J Clin Invest.

[21] Jabri B, Sollid LM (2017). T cells in celiac disease. J Immunol.

[22] Zimmermann C, Rudloff S, Lochnit G, Arampatzi S, Maison W, Zimmer KP (2014). Epithelial transport of immunogenic and toxic gliadin peptides in vitro. PLoS ONE.

[23] Shan L, Molberg O, Parrot I, Hausch F, Filiz F, Gray GM, Sollid LM, Khosla C (2002). Structural basis for gluten intolerance in celiac sprue. Science.

[24] Fasano A (2011). Zonulin and its regulation of intestinal barrier function: the biological door to inflammation, autoimmunity, and cancer. Physiol Rev.

[25] Drago S, El Asmar R, Di Pierro M, Grazia Clemente M, Tripathi A, Sapone A, Thakar M, Iacono G, Carroccio A, D'Agate C, Not T, Zampini L, Catassi C, Fasano A (2006). Gliadin, zonulin and gut permeability: effects on celiac and non-celiac intestinal mucosa and intestinal cell lines. Scand J Gastroenterol.

[26] Tripathi A, Lammers KM, Goldblum S, Shea-Donohue T, Netzel-Arnett S, Buzza MS, Antalis TM, Vogel SN, Zhao A, Yang S, Arrietta M-C, Meddings JB, Fasano A (2009). Identification of human zonulin, a physiological modulator of tight junctions, as prehaptoglobin-2. Proc Natl Acad Sci USA.

[27] Molberg O, McAdam SN, Korner R, Quarsten H, Kristiansen C, Madsen L, Fugger L, Scott H, Noren O, Roepstorff P, Lundin KE, Sjostrom H, Sollid LM (1998). Tissue transglutaminase selectively modifies gliadin peptides that are recognized by gut-derived T cells in celiac disease. Nat Med.

[28] Sollid LM, Markussen G, Ek J, Gjerde H, Vartdal F, Thorsby E (1989). Evidence for a primary association of celiac disease to a particular HLA-DQ alpha/beta heterodimer. J Exp Med.

[29] Karell K, Louka AS, Moodie SJ, Ascher H, Clot F, Greco L, Ciclitira PJ, Sollid LM, Partanen J, European Genetics Cluster on Celiac D (2003). HLA types in celiac disease patients not carrying the DQA1*05-DQB1*02 (DQ2) heterodimer: results from the European genetics cluster on celiac disease. Hum Immunol.

[30] Meresse B, Malamut G, Cerf-Bensussan N (2012). Celiac disease: an immunological jigsaw. Immunity.

[31] Yu XB, Uhde M, Green PH, Alaedini A (2018). Autoantibodies in the extraintestinal manifestations of celiac disease. Nutrients.

[32] Di Sabatino A, Corazza GR (2009). Coeliac disease. Lancet.

[33] van Leeuwen MA, Lindenbergh-Kortleve DJ, Raatgeep HC, de Ruiter LF, de Krijger RR, Groeneweg M, Escher JC, Samsom JN (2013). Increased production of interleukin-21, but not interleukin-17A, in the small intestine characterizes pediatric celiac disease. Mucosal Immunol.

[34] Abadie V, Jabri B (2014). IL-15: a central regulator of celiac disease immunopathology. Immunol Rev.

[35] Uhrberg M, Valiante NM, Young NT, Lanier LL, Phillips JH, Parham P (2001). The repertoire of killer cell Ig-like receptor and CD94:NKG2A receptors in T cells: clones sharing identical alpha beta TCR rearrangement express highly diverse killer cell Ig-like receptor patterns. J Immunol.

[36] Jabri B, Sollid LM (2009). Tissue-mediated control of immunopathology in coeliac disease. Nat Rev Immunol.

[37] Thome JJ, Farber DL (2015). Emerging concepts in tissue-resident T cells: lessons from humans. Trends Immunol.

[38] Sarra M, Cupi ML, Monteleone I, Franze E, Ronchetti G, Di Sabatino A, Gentileschi P, Franceschilli L, Sileri P, Sica G, Del Vecchio BG, Cretella M, Paoluzi OA, Corazza GR, Pallone F, Monteleone G (2013). IL-15 positively regulates IL-21 production in celiac disease mucosa. Mucosal Immunol.

[39] Ciccocioppo R, Di Sabatino A, Corazza GR (2005). The immune recognition of gluten in coeliac disease. Clin Exp Immunol.

[40] Foundation Cd future therapies for celiac disease. https://celiac.org/about-celiac-disease/future-therapies-for-celiac-disease/

[41] Withoff S, Li Y, Jonkers I, Wijmenga C (2016). Understanding celiac disease by genomics. Trends Genet.

[42] Catassi C, Kryszak D, Bhatti B, Sturgeon C, Helzlsouer K, Clipp SL, Gelfond D, Puppa E, Sferruzza A, Fasano A (2010). Natural history of celiac disease autoimmunity in a USA cohort followed since 1974. Ann Med.

[43] Vriezinga SL, Auricchio R, Bravi E, Castillejo G, Chmielewska A, Crespo Escobar P, Kolaček S, Koletzko S, Korponay-Szabo IR, Mummert E, Polanco I, Putter H, Ribes-Koninckx C, Shamir R, Szajewska H, Werkstetter K, Greco L, Gyimesi J, Hartman C, Hogen Esch C, Hopman E, Ivarsson A, Koltai T, Koning F, Martinez-Ojinaga E, te Marvelde C, Pavic A, Romanos J, Stoopman E, Villanacci V, Wijmenga C, Troncone R, Mearin ML (2014). Randomized feeding intervention in infants at high risk for celiac disease. N Engl J Med.

[44] Farrell RJ, Kelly CP (2002). Celiac sprue. N Engl J Med.

[45] Lammers KM, Herrera MG, Dodero VI (2018). Translational chemistry meets gluten-related disorders. Chemist Open.

[46] Ni J, Wu GD, Albenberg L, Tomov VT (2017). Gut microbiota and IBD: causation or correlation?. Nat Rev Gastroenterol Hepatol.

[47] Maruvada P, Leone V, Kaplan LM, Chang EB (2017). The human microbiome and obesity: moving beyond associations. Cell Host Microbe.

[48] Turnbaugh PJ, Ley RE, Mahowald MA, Magrini V, Mardis ER, Gordon JI (2006). An obesity-associated gut microbiome with increased capacity for energy harvest. Nature.

[49] Jandhyala SM, Talukdar R, Subramanyam C, Vuyyuru H, Sasikala M, Nageshwar Reddy D (2015). Role of the normal gut microbiota. World J Gastroenterol.

[50] Backhed F, Ley RE, Sonnenburg JL, Peterson DA, Gordon JI (2005). Host-bacterial mutualism in the human intestine. Science.

[51] Savage DC (1977). Microbial ecology of the gastrointestinal tract. Annu Rev Microbiol.

[52] Singh P, Kumar M, Al Khodor S (2019). Vitamin D deficiency in the gulf cooperation council: exploring the triad of genetic predisposition, the gut microbiome and the immune system. Front Immunol.

[53] Shapira M (2016). Gut microbiotas and host evolution: scaling up symbiosis. Trends Ecol Evol.

[54] Rodriguez JM, Murphy K, Stanton C, Ross RP, Kober OI, Juge N, Avershina E, Rudi K, Narbad A, Jenmalm MC, Marchesi JR, Collado MC (2015). The composition of the gut microbiota throughout life, with an emphasis on early life. Microb Ecol Health Dis.

[55] Funkhouser LJ, Bordenstein SR (2013). Mom knows best: the universality of maternal microbial transmission. PLoS Biol.

[56] Fouhy F, Watkins C, Hill CJ, O’Shea CA, Nagle B, Dempsey EM, O'Toole PW, Ross RP, Ryan CA, Stanton C (2019). Perinatal factors affect the gut microbiota up to four years after birth. Nat Commun.

[57] Quigley EMM (2013). Gut bacteria in health and disease. Gastroenterolo Hepatol.

[58] Clarke G, Stilling RM, Kennedy PJ, Stanton C, Cryan JF, Dinan TG (2014). Minireview: gut microbiota: the neglected endocrine organ. Mol Endocrinol.

[59] Hutchison CA (2007). DNA sequencing: bench to bedside and beyond. Nucleic Acids Res.

[60] Rawat A, Engelthaler DM, Driebe EM, Keim P, Foster JT (2014). MetaGeniE: characterizing human clinical samples using deep metagenomic sequencing. PLoS ONE.

[61] Scheuch M, Höper D, Beer M (2015). RIEMS: a software pipeline for sensitive and comprehensive taxonomic classification of reads from metagenomics datasets. BMC Bioinform.

[62] Human Microbiome Project C (2012). Structure, function and diversity of the healthy human microbiome. Nature.

[63] Rautava S, Luoto R, Salminen S, Isolauri E (2012). Microbial contact during pregnancy, intestinal colonization and human disease. Nat Rev Gastroenterol Hepatol.

[64] Cheng J, Kalliomaki M, Heilig HG, Palva A, Lahteenoja H, de Vos WM, Salojarvi J, Satokari R (2013). Duodenal microbiota composition and mucosal homeostasis in pediatric celiac disease. BMC Gastroenterol.

[65] Wacklin P, Kaukinen K, Tuovinen E, Collin P, Lindfors K, Partanen J, Maki M, Matto J (2013). The duodenal microbiota composition of adult celiac disease patients is associated with the clinical manifestation of the disease. Inflamm Bowel Dis.

[66] O'Hara AM, Shanahan F (2006). The gut flora as a forgotten organ. EMBO Rep.

[67] Ensari A (2010). Gluten-sensitive enteropathy (celiac disease): controversies in diagnosis and classification. Arch Pathol Lab Med.

[68] Rondonotti E, Spada C, Cave D, Pennazio M, Riccioni ME, De Vitis I, Schneider D, Sprujevnik T, Villa F, Langelier J, Arrigoni A, Costamagna G, de Franchis R (2007). Video capsule enteroscopy in the diagnosis of celiac disease: a multicenter study. Am J Gastroenterol.

[69] Nurminen S, Kivelä L, Huhtala H, Kaukinen K, Kurppa K (2019). Extraintestinal manifestations were common in children with coeliac disease and were more prevalent in patients with more severe clinical and histological presentation. Acta Paediatr.

[70] De Palma G, Nadal I, Collado MC, Sanz Y (2009). Effects of a gluten-free diet on gut microbiota and immune function in healthy adult human subjects. Br J Nutr.

[71] De Palma G, Nadal I, Medina M, Donat E, Ribes-Koninckx C, Calabuig M, Sanz Y (2010). Intestinal dysbiosis and reduced immunoglobulin-coated bacteria associated with coeliac disease in children. BMC Microbiol.

[72] De Palma G, Capilla A, Nadal I, Nova E, Pozo T, Varea V, Polanco I, Castillejo G, Lopez A, Garrote JA, Calvo C, Garcia-Novo MD, Cilleruelo ML, Ribes-Koninckx C, Palau F, Sanz Y (2010). Interplay between human leukocyte antigen genes and the microbial colonization process of the newborn intestine. Curr Issues Mol Biol.

[73] Nadal I, Santacruz A, Marcos A, Warnberg J, Garagorri JM, Moreno LA, Martin-Matillas M, Campoy C, Marti A, Moleres A, Delgado M, Veiga OL, Garcia-Fuentes M, Redondo CG, Sanz Y (2009). Shifts in clostridia, bacteroides and immunoglobulin-coating fecal bacteria associated with weight loss in obese adolescents. Int J Obes (Lond).

[74] Di Cagno R, De Angelis M, De Pasquale I, Ndagijimana M, Vernocchi P, Ricciuti P, Gagliardi F, Laghi L, Crecchio C, Guerzoni ME, Gobbetti M, Francavilla R (2011). Duodenal and faecal microbiota of celiac children: molecular, phenotype and metabolome characterization. BMC Microbiol.

[75] Collado MC, Calabuig M, Sanz Y (2007). Differences between the fecal microbiota of coeliac infants and healthy controls. Curr Issues Intest Microbiol.

[76] Collado MC, Donat E, Ribes-Koninckx C, Calabuig M, Sanz Y (2008). Imbalances in faecal and duodenal Bifidobacterium species composition in active and non-active coeliac disease. BMC Microbiol.

[77] Collado MC, Donat E, Ribes-Koninckx C, Calabuig M, Sanz Y (2009). Specific duodenal and faecal bacterial groups associated with paediatric coeliac disease. J Clin Pathol.

[78] Nistal E, Caminero A, Herran AR, Arias L, Vivas S, de Morales JM, Calleja S, de Miera LE, Arroyo P, Casqueiro J (2012). Differences of small intestinal bacteria populations in adults and children with/without celiac disease: effect of age, gluten diet, and disease. Inflamm Bowel Dis.

[79] Schippa S, Iebba V, Barbato M, Di Nardo G, Totino V, Checchi MP, Longhi C, Maiella G, Cucchiara S, Conte MP (2010). A distinctive ‘microbial signature’ in celiac pediatric patients. BMC Microbiol.

[80] Sanchez E, Donat E, Ribes-Koninckx C, Calabuig M, Sanz Y (2010). Intestinal bacteroides species associated with coeliac disease. J Clin Pathol.

[81] Sánchez E, Donat E, Ribes-Koninckx C, Fernández-Murga ML, Sanz Y (2013). Duodenal-mucosal bacteria associated with celiac disease in children. Appl Environ Microbiol.

[82] Bodkhe R, Shetty SA, Dhotre DP, Verma AK, Bhatia K, Mishra A, Kaur G, Pande P, Bangarusamy DK, Santosh BP, Perumal RC, Ahuja V, Shouche YS, Makharia GK (2019). Comparison of small gut and whole gut microbiota of first-degree relatives with adult celiac disease patients and controls. Front Microbiol.

[83] Golfetto L, de Senna FD, Hermes J, Beserra BT, Franca Fda S, Martinello F (2014). Lower bifidobacteria counts in adult patients with celiac disease on a gluten-free diet. Arq Gastroenterol.

[84] Wacklin P, Laurikka P, Lindfors K, Collin P, Salmi T, Lahdeaho ML, Saavalainen P, Maki M, Matto J, Kurppa K, Kaukinen K (2014). Altered duodenal microbiota composition in celiac disease patients suffering from persistent symptoms on a long-term gluten-free diet. Am J Gastroenterol.

[85] Garcia-Mazcorro JF, Rivera-Gutierrez X, Cobos-Quevedo OJ, Grube-Pagola P, Meixueiro-Daza A, Hernandez-Flores K, Cabrera-Jorge FJ, Vivanco-Cid H, Dowd SE, Remes-Troche JM (2018). First insights into the gut microbiota of mexican patients with celiac disease and non-celiac gluten sensitivity. Nutrients.

[86] Verdu EF, Galipeau HJ, Jabri B (2015). Novel players in coeliac disease pathogenesis: role of the gut microbiota. Nat Rev Gastroenterol Hepatol.

[87] Forsberg G, Fahlgren A, Hörstedt P, Hammarström S, Hernell O, Hammarström M-L (2004). Presence of bacteria and innate immunity of intestinal epithelium in childhood celiac disease. Am J Gastroenterol.

[88] Ou G, Hedberg M, Hörstedt P, Baranov V, Forsberg G, Drobni M, Sandström O, Wai SN, Johansson I, Hammarström M-L, Hernell O, Hammarström S (2009). Proximal small intestinal microbiota and identification of rod-shaped bacteria associated with childhood celiac disease. Am J Gastroenterol.

[89] Sanchez E, Donat E, Ribes-Koninckx C, Fernandez-Murga ML, Sanz Y (2013). Duodenal-mucosal bacteria associated with celiac disease in children. Appl Environ Microbiol.

[90] Sanz Y, Sanchez E, Marzotto M, Calabuig M, Torriani S, Dellaglio F (2007). Differences in faecal bacterial communities in coeliac and healthy children as detected by PCR and denaturing gradient gel electrophoresis. FEMS Immunol Med Microbiol.

[91] Di Cagno R, Rizzello CG, Gagliardi F, Ricciuti P, Ndagijimana M, Francavilla R, Guerzoni ME, Crecchio C, Gobbetti M, De Angelis M (2009). Different fecal microbiotas and volatile organic compounds in treated and untreated children with celiac disease. Appl Environ Microbiol.

[92] De Palma G, Nadal I, Medina M, Donat E, Ribes-Koninckx C, Calabuig M, Sanz Y (2010). Intestinal dysbiosis and reduced immunoglobulin-coated bacteria associated with coeliac disease in children. BMC Microbiol.

[93] Olivares M, Walker AW, Capilla A, Benítez-Páez A, Palau F, Parkhill J, Castillejo G, Sanz Y (2018). Gut microbiota trajectory in early life may predict development of celiac disease. Microbiome.

[94] Shiba T, Aiba Y, Ishikawa H, Ushiyama A, Takagi A, Mine T, Koga Y (2003). The suppressive effect of bifidobacteria on Bacteroides vulgatus, a putative pathogenic microbe in inflammatory bowel disease. Microbiol Immunol.

[95] Sydora BC, Macfarlane SM, Walker JW, Dmytrash AL, Churchill TA, Doyle J, Fedorak RN (2007). Epithelial barrier disruption allows nondisease-causing bacteria to initiate and sustain IBD in the IL-10 gene-deficient mouse. Inflamm Bowel Dis.

[96] Setoyama H, Imaoka A, Ishikawa H, Umesaki Y (2003). Prevention of gut inflammation by *Bifidobacterium* in dextran sulfate-treated gnotobiotic mice associated with *Bacteroides* strains isolated from ulcerative colitis patients. Microbes Infect.

[97] Nistal E, Caminero A, Vivas S, Ruiz de Morales JM, Saenz de Miera LE, Rodriguez-Aparicio LB, Casqueiro J (2012). Differences in faecal bacteria populations and faecal bacteria metabolism in healthy adults and celiac disease patients. Biochimie.

[98] Caminero A, Galipeau HJ, McCarville JL, Johnston CW, Bernier SP, Russell AK, Jury J, Herran AR, Casqueiro J, Tye-Din JA, Surette MG, Magarvey NA, Schuppan D, Verdu EF (2016). Duodenal bacteria from patients with celiac disease and healthy subjects distinctly affect gluten breakdown and immunogenicity. Gastroenterology.

[99] Olivares M, Neef A, Castillejo G, Palma GD, Varea V, Capilla A, Palau F, Nova E, Marcos A, Polanco I, Ribes-Koninckx C, Ortigosa L, Izquierdo L, Sanz Y (2015). The HLA-DQ2 genotype selects for early intestinal microbiota composition in infants at high risk of developing coeliac disease. Gut.

[100] Sellitto M, Bai G, Serena G, Fricke WF, Sturgeon C, Gajer P, White JR, Koenig SS, Sakamoto J, Boothe D, Gicquelais R, Kryszak D, Puppa E, Catassi C, Ravel J, Fasano A (2012). Proof of concept of microbiome-metabolome analysis and delayed gluten exposure on celiac disease autoimmunity in genetically at-risk infants. PLoS ONE.

[101] Di Cagno R, De Angelis M, Lavermicocca P, De Vincenzi M, Giovannini C, Faccia M, Gobbetti M (2002). Proteolysis by sourdough lactic acid bacteria: effects on wheat flour protein fractions and gliadin peptides involved in human cereal intolerance. Appl Environ Microbiol.

[102] Zamakhchari M, Wei G, Dewhirst F, Lee J, Schuppan D, Oppenheim FG, Helmerhorst EJ (2011). Identification of Rothia bacteria as gluten-degrading natural colonizers of the upper gastro-intestinal tract. PLoS ONE.

[103] Chen VL, Kasper DL (2014). Interactions between the intestinal microbiota and innate lymphoid cells. Gut Microbes.

[104] Moro K, Koyasu S (2015). Innate lymphoid cells, possible interaction with microbiota. Semin Immunopathol.

[105] Hrncir T, Stepankova R, Kozakova H, Hudcovic T, Tlaskalova-Hogenova H (2008). Gut microbiota and lipopolysaccharide content of the diet influence development of regulatory T cells: studies in germ-free mice. BMC Immunol.

[106] Arpaia N, Campbell C, Fan X, Dikiy S, van der Veeken J, deRoos P, Liu H, Cross JR, Pfeffer K, Coffer PJ, Rudensky AY (2013). Metabolites produced by commensal bacteria promote peripheral regulatory T-cell generation. Nature.

[107] Furusawa Y, Obata Y, Fukuda S, Endo TA, Nakato G, Takahashi D, Nakanishi Y, Uetake C, Kato K, Kato T, Takahashi M, Fukuda NN, Murakami S, Miyauchi E, Hino S, Atarashi K, Onawa S, Fujimura Y, Lockett T, Clarke JM, Topping DL, Tomita M, Hori S, Ohara O, Morita T, Koseki H, Kikuchi J, Honda K, Hase K, Ohno H (2013). Commensal microbe-derived butyrate induces the differentiation of colonic regulatory T cells. Nature.

[108] Smith PM, Howitt MR, Panikov N, Michaud M, Gallini CA, Bohlooly YM, Glickman JN, Garrett WS (2013). The microbial metabolites, short-chain fatty acids, regulate colonic Treg cell homeostasis. Science.

[109] Wylie KM, Weinstock GM, Storch GA (2012). Emerging view of the human virome. Transl Res.

[110] Lerner A, Matthias T, Aminov R (2017). Potential effects of horizontal gene exchange in the human gut. Front Immunol.

[111] Mills S, Shanahan F, Stanton C, Hill C, Coffey A, Ross RP (2013). Movers and shakers: influence of bacteriophages in shaping the mammalian gut microbiota. Gut Microbes.

[112] Navarro F, Muniesa M (2017). Phages in the Human Body. Front Microbiol.

[113] Manrique P, Dills M, Young MJ (2017). The human gut phage community and its implications for health and disease. Viruses.

[114] Chopin MC, Chopin A, Bidnenko E (2005). Phage abortive infection in lactococci: variations on a theme. Curr Opin Microbiol.

[115] Kagnoff MF, Paterson YJ, Kumar PJ, Kasarda DD, Carbone FR, Unsworth DJ, Austin RK (1987). Evidence for the role of a human intestinal adenovirus in the pathogenesis of coeliac disease. Gut.

[116] Stene LC, Honeyman MC, Hoffenberg EJ, Haas JE, Sokol RJ, Emery L, Taki I, Norris JM, Erlich HA, Eisenbarth GS, Rewers M (2006). Rotavirus infection frequency and risk of celiac disease autoimmunity in early childhood: a longitudinal study. Am J Gastroenterol.

[117] Myléus A, Hernell O, Gothefors L, Hammarström M-L, Persson L-Å, Stenlund H, Ivarsson A (2012). Early infections are associated with increased risk for celiac disease: an incident case-referent study. BMC Pediat.

[118] Kemppainen KM, Lynch KF, Liu E, Lonnrot M, Simell V, Briese T, Koletzko S, Hagopian W, Rewers M, She JX, Simell O, Toppari J, Ziegler AG, Akolkar B, Krischer JP, Lernmark A, Hyoty H, Triplett EW, Agardh D, Group TS (2017). Factors that increase risk of celiac disease autoimmunity after a gastrointestinal infection in early life. Clin Gastroenterol Hepatol.

[119] Ziberna F, De Lorenzo G, Schiavon V, Arnoldi F, Quaglia S, De Leo L, Vatta S, Martelossi S, Burrone OR, Ventura A, Not T (2016). Lack of evidence of rotavirus-dependent molecular mimicry as a trigger of coeliac disease. Clin Exp Immunol.

[120] Plot L, Amital H (2009). Infectious associations of Celiac disease. Autoimmun Rev.

[121] Bouziat R, Hinterleitner R, Brown JJ, Stencel-Baerenwald JE, Ikizler M, Mayassi T, Meisel M, Kim SM, Discepolo V, Pruijssers AJ, Ernest JD, Iskarpatyoti JA, Costes LM, Lawrence I, Palanski BA, Varma M, Zurenski MA, Khomandiak S, McAllister N, Aravamudhan P, Boehme KW, Hu F, Samsom JN, Reinecker HC, Kupfer SS, Guandalini S, Semrad CE, Abadie V, Khosla C, Barreiro LB, Xavier RJ, Ng A, Dermody TS, Jabri B (2017). Reovirus infection triggers inflammatory responses to dietary antigens and development of celiac disease. Science.

[122] Kahrs CR, Chuda K, Tapia G, Stene LC, Mårild K, Rasmussen T, Rønningen KS, Lundin KEA, Kramna L, Cinek O, Størdal K (2019). Enterovirus as trigger of coeliac disease: nested case-control study within prospective birth cohort. BMJ.

[123] Lindfors K, Lin J, Lee H-S, Hyöty H, Nykter M, Kurppa K, Liu E, Koletzko S, Rewers M, Hagopian W, Toppari J, Ziegler A-G, Akolkar B, Krischer JP, Petrosino JF, Lloyd RE, Agardh D (2019). Metagenomics of the faecal virome indicate a cumulative effect of enterovirus and gluten amount on the risk of coeliac disease autoimmunity in genetically at risk children: the TEDDY study. Gut.

[124] Caminero A, Herrán AR, Nistal E, Pérez-Andrés J, Vaquero L, Vivas S, Ruiz de Morales JMG, Albillos SM, Casqueiro J (2014). Diversity of the cultivable human gut microbiome involved in gluten metabolism: isolation of microorganisms with potential interest for coeliac disease. FEMS Microbiol Ecol.

[125] Costello EK, Lauber CL, Hamady M, Fierer N, Gordon JI, Knight R (2009). Bacterial community variation in human body habitats across space and time. Science (New York, NY).

[126] Curtis MA, Zenobia C, Darveau RP (2011). The relationship of the oral microbiotia to periodontal health and disease. Cell Host Microbe.

[127] Wieser H (1995). 1 The precipitating factor in coeliac disease. Bailliere’s Clin Gastroenterol.

[128] Shan L, Molberg Ø, Parrot I, Hausch F, Filiz F, Gray GM, Sollid LM, Khosla C (2002). Structural basis for gluten intolerance in celiac sprue. Science (New York, NY).

[129] Vader LW, de Ru A, van der Wal Y, Kooy YM, Benckhuijsen W, Mearin ML, Drijfhout JW, van Veelen P, Koning F (2002). Specificity of tissue transglutaminase explains cereal toxicity in celiac disease. J Exp Med.

[130] Helmerhorst EJ, Zamakhchari M, Schuppan D, Oppenheim FG (2010). Discovery of a novel and rich source of gluten-degrading microbial enzymes in the oral cavity. PLoS ONE.

[131] Francavilla R, Ercolini D, Piccolo M, Vannini L, Siragusa S, De Filippis F, De Pasquale I, Di Cagno R, Di Toma M, Gozzi G, Serrazanetti DI, De Angelis M, Gobbetti M (2014). Salivary microbiota and metabolome associated with celiac disease. Appl Environ Microbiol.

[132] Tian N, Faller L, Leffler DA, Kelly CP, Hansen J, Bosch JA, Wei G, Paster BJ, Schuppan D, Helmerhorst EJ (2017). Salivary gluten degradation and oral microbial profiles in healthy individuals and celiac disease patients. Appl Environ Microbiol.

[133] Iaffaldano L, Granata I, Pagliuca C, Esposito MV, Casaburi G, Salerno G, Colicchio R, Piccirillo M, Ciacci C, Del Vecchio BG, Guarracino MR, Salvatore P, Salvatore F, D’Argenio V, Sacchetti L (2018). Oropharyngeal microbiome evaluation highlights Neisseria abundance in active celiac patients. Scientific Reports.

[134] Païssé S, Valle C, Servant F, Courtney M, Burcelin R, Amar J, Lelouvier B (2016). Comprehensive description of blood microbiome from healthy donors assessed by 16 S targeted metagenomic sequencing. Transfusion.

[135] Potgieter M, Bester J, Kell DB, Pretorius E (2015). The dormant blood microbiome in chronic, inflammatory diseases. FEMS Microbiol Rev.

[136] Proal AD, Albert PJ, Marshall TG (2014). Inflammatory disease and the human microbiome. Discov Med.

[137] Castillo DJ, Rifkin RF, Cowan DA, Potgieter M (2019). The Healthy Human Blood Microbiome: Fact or Fiction?. Front Cell Infect Microbiol.

[138] Amar J, Lange C, Payros G, Garret C, Chabo C, Lantieri O, Courtney M, Marre M, Charles MA, Balkau B (2013) Blood microbiota dysbiosis is associated with the onset of cardiovascular events in a large general population: the DESIR study. PLoS One 8(1). 10.1371/journal.pone.0054461 10.1371/journal.pone.0054461 PMC3555817 23372728

[139] Sato J, Kanazawa A, Ikeda F, Yoshihara T, Goto H, Abe H, Komiya K, Kawaguchi M, Shimizu T, Ogihara T (2014). Gut dysbiosis and detection of “live gut bacteria” in blood of Japanese patients with type 2 diabetes. Diabetes Care.

[140] Serena G, Davies C, Cetinbas M, Sadreyev RI, Fasano A (2019). Analysis of blood and fecal microbiome profile in patients with celiac disease. Human Microb J.

[141] Galipeau HJ, Verdu EF (2014). Gut microbes and adverse food reactions: focus on gluten related disorders. Gut Microbes.

[142] Lebwohl B, Ludvigsson JF, Green PH (2015). Celiac disease and non-celiac gluten sensitivity. BMJ.

[143] Rossi M, Schwartz KB (2010). Editorial: celiac disease and intestinal bacteria: not only gluten?. J Leukoc Biol.

[144] Dominguez-Bello MG, Costello EK, Contreras M, Magris M, Hidalgo G, Fierer N, Knight R (2010). Delivery mode shapes the acquisition and structure of the initial microbiota across multiple body habitats in newborns. Proc Natl Acad Sci.

[145] Salminen S, Gibson GR, McCartney AL, Isolauri E (2004). Influence of mode of delivery on gut microbiota composition in seven year old children. Gut.

[146] Decker E, Engelmann G, Findeisen A, Gerner P, Laass M, Ney D, Posovszky C, Hoy L, Hornef MW (2010). Cesarean delivery is associated with celiac disease but not inflammatory bowel disease in children. Pediatrics.

[147] Emilsson L, Magnus MC, Stordal K (2015). Perinatal risk factors for development of celiac disease in children, based on the prospective norwegian mother and child cohort study. Clin Gastroenterol Hepatol.

[148] Namatovu F, Olsson C, Lindkvist M, Myléus A, Högberg U, Ivarsson A, Sandström O (2016). Maternal and perinatal conditions and the risk of developing celiac disease during childhood. BMC Pediat.

[149] Mårild K, Stephansson O, Montgomery S, Murray JA, Ludvigsson JF (2012). Pregnancy outcome and risk of celiac disease in offspring: a nationwide case-control study. Gastroenterology.

[150] Canova C, Zabeo V, Pitter G, Romor P, Baldovin T, Zanotti R, Simonato L (2014). Association of maternal education, early infections, and antibiotic use with celiac disease: a population-based birth cohort study in northeastern Italy. Am J Epidemiol.

[151] Becattini S, Taur Y, Pamer EG (2016). Antibiotic-induced changes in the intestinal microbiota and disease. Trends Mol Med.

[152] Ianiro G, Tilg H, Gasbarrini A (2016). Antibiotics as deep modulators of gut microbiota: between good and evil. Gut.

[153] Bokulich NA, Chung J, Battaglia T, Henderson N, Jay M, Li H, DL A, Wu F, Perez-Perez GI, Chen Y, Schweizer W, Zheng X, Contreras M, Dominguez-Bello MG, Blaser MJ (2016). Antibiotics, birth mode, and diet shape microbiome maturation during early life. Sci Transl Med.

[154] Pozo-Rubio T, de Palma G, Mujico JR, Olivares M, Marcos A, Acuña MD, Polanco I, Sanz Y, Nova E (2013). Influence of early environmental factors on lymphocyte subsets and gut microbiota in infants at risk of celiac disease; the PROFICEL study. Nutr Hosp.

[155] Dydensborg Sander S, Nybo Andersen AM, Murray JA, Karlstad O, Husby S, Stordal K (2019). Association Between Antibiotics in the First Year of Life and Celiac Disease. Gastroenterology.

[156] Marild K, Ye W, Lebwohl B, Green PH, Blaser MJ, Card T, Ludvigsson JF (2013). Antibiotic exposure and the development of coeliac disease: a nationwide case-control study. BMC Gastroenterol.

[157] Bittker SS, Bell KR (2019). Potential risk factors for celiac disease in childhood: a case-control epidemiological survey. Clin Exp Gastroenterol.

[158] Bennett BJ, Hall KD, Hu FB, McCartney AL, Roberto C (2015). Nutrition and the science of disease prevention: a systems approach to support metabolic health. Ann N Y Acad Sci.

[159] Ordovas JM, Ferguson LR, Tai ES, Mathers JC (2018). Personalised nutrition and health. BMJ.

[160] Loos RJF (2019). From nutrigenomics to personalizing diets: are we ready for precision medicine?. Am J Clin Nutr.

[161] Macready AL, Fallaize R, Butler LT, Ellis JA, Kuznesof S, Frewer LJ, Celis-Morales C, Livingstone KM, Araujo-Soares V, Fischer AR, Stewart-Knox BJ, Mathers JC, Lovegrove JA (2018). Application of behavior change techniques in a personalized nutrition electronic health intervention study: protocol for the web-based food4me randomized controlled trial. JMIR Res Protoc.

[162] Singh RK, Chang HW, Yan D, Lee KM, Ucmak D, Wong K, Abrouk M, Farahnik B, Nakamura M, Zhu TH, Bhutani T, Liao W (2017). Influence of diet on the gut microbiome and implications for human health. J Transl Med.

[163] Schmidt TSB, Raes J, Bork P (2018). The human gut microbiome: from association to modulation. Cell.

[164] Maier L, Pruteanu M, Kuhn M, Zeller G, Telzerow A, Anderson EE, Brochado AR, Fernandez KC, Dose H, Mori H, Patil KR, Bork P, Typas A (2018). Extensive impact of non-antibiotic drugs on human gut bacteria. Nature.

[165] Kumar M, Mathur T, Joshi V, Upadhyay DJ, Inoue SI, Masuda N (2018). Effect of DS-2969b, a novel GyrB inhibitor, on rat and monkey intestinal microbiota. Anaerobe.

[166] Dudek-Wicher RK, Junka A, Bartoszewicz M (2018). The influence of antibiotics and dietary components on gut microbiota. Prz Gastroenterol.

[167] Statovci D, Aguilera M, MacSharry J, Melgar S (2017). The impact of western diet and nutrients on the microbiota and immune response at mucosal interfaces. Front Immunol.

[168] David LA, Maurice CF, Carmody RN, Gootenberg DB, Button JE, Wolfe BE, Ling AV, Devlin AS, Varma Y, Fischbach MA, Biddinger SB, Dutton RJ, Turnbaugh PJ (2014). Diet rapidly and reproducibly alters the human gut microbiome. Nature.

[169] Pinzone MR, Celesia BM, Di Rosa M, Cacopardo B, Nunnari G (2012). Microbial translocation in chronic liver diseases. Int J Microbiol.

[170] World Health Organization (2003) Diet, nutrition, and the prevention of chronic diseases: report of a joint WHO/FAO expert consultation 916. https://apps.who.int/iris/bitstream/handle/10665/42665/WHO_TRS_916.pdf;jsessionid=4D311C3BE1D830F26EDDA65210DE5A22?sequence=1 12768890

[171] Akobeng AK, Thomas AG (2008). Systematic review: tolerable amount of gluten for people with coeliac disease. Aliment Pharmacol Ther.

[172] Ciacci C, Ciclitira P, Hadjivassiliou M, Kaukinen K, Ludvigsson JF, McGough N, Sanders DS, Woodward J, Leonard JN, Swift GL (2015). The gluten-free diet and its current application in coeliac disease and dermatitis herpetiformis. United Eur Gastroenterol J.

[173] Hogberg L, Grodzinsky E, Stenhammar L (2003). Better dietary compliance in patients with coeliac disease diagnosed in early childhood. Scand J Gastroenterol.

[174] De Palma G, Nadal I, Collado MC, Sanz Y (2009). Effects of a gluten-free diet on gut microbiota and immune function in healthy adult human subjects. Br J Nutr.

[175] Jackson FW (2010). Effects of a gluten-free diet on gut microbiota and immune function in healthy adult human subjects - comment by Jackson. Br J Nutr.

[176] Sarin SK, Pande A, Schnabl B (2019). Microbiome as a therapeutic target in alcohol-related liver disease. J Hepatol.

[177] Khoruts A (2018). Targeting the microbiome: from probiotics to fecal microbiota transplantation. Gen Med.

[178] Roncoroni L, Bascunan KA, Doneda L, Scricciolo A, Lombardo V, Branchi F, Ferretti F, Dell'Osso B, Montanari V, Bardella MT, Elli L (2018). A low fodmap gluten-free diet improves functional gastrointestinal disorders and overall mental health of celiac disease patients: a randomized controlled trial. Nutrients.

[179] Reddel S, Putignani L, Del Chierico F (2019). The impact of low-FODMAPs, gluten-free, and ketogenic diets on gut microbiota modulation in pathological conditions. Nutrients.

[180] Dieterich W, Schuppan D, Schink M, Schwappacher R, Wirtz S, Agaimy A, Neurath MF, Zopf Y (2019). Influence of low FODMAP and gluten-free diets on disease activity and intestinal microbiota in patients with non-celiac gluten sensitivity. Clin Nutr.

[181] Vanderpool C, Yan F, Polk DB (2008). Mechanisms of probiotic action: Implications for therapeutic applications in inflammatory bowel diseases. Inflamm Bowel Dis.

[182] Laparra JM, Sanz Y (2010). Bifidobacteria inhibit the inflammatory response induced by gliadins in intestinal epithelial cells via modifications of toxic peptide generation during digestion. J Cell Biochem.

[183] Lindfors K, Blomqvist T, Juuti-Uusitalo K, Stenman S, Venalainen J, Maki M, Kaukinen K (2008). Live probiotic Bifidobacterium lactis bacteria inhibit the toxic effects induced by wheat gliadin in epithelial cell culture. Clin Exp Immunol.

[184] Smecuol E, Hwang HJ, Sugai E, Corso L, Chernavsky AC, Bellavite FP, Gonzalez A, Vodanovich F, Moreno ML, Vazquez H, Lozano G, Niveloni S, Mazure R, Meddings J, Maurino E, Bai JC (2013). Exploratory, randomized, double-blind, placebo-controlled study on the effects of Bifidobacterium infantis natren life start strain super strain in active celiac disease. J Clin Gastroenterol.

[185] Olivares M, Castillejo G, Varea V, Sanz Y (2014). Double-blind, randomised, placebo-controlled intervention trial to evaluate the effects of Bifidobacterium longum CECT 7347 in children with newly diagnosed coeliac disease. Br J Nutr.

[186] Di Cagno R, De Angelis M, Auricchio S, Greco L, Clarke C, De Vincenzi M, Giovannini C, D'Archivio M, Landolfo F, Parrilli G, Minervini F, Arendt E, Gobbetti M (2004). Sourdough bread made from wheat and nontoxic flours and started with selected lactobacilli is tolerated in celiac sprue patients. Appl Environ Microbiol.

[187] Greco L, Gobbetti M, Auricchio R, Di Mase R, Landolfo F, Paparo F, Di Cagno R, De Angelis M, Rizzello CG, Cassone A, Terrone G, Timpone L, D'Aniello M, Maglio M, Troncone R, Auricchio S (2011). Safety for patients with celiac disease of baked goods made of wheat flour hydrolyzed during food processing. Clin Gastroenterol Hepatol.

[188] De Angelis M, Rizzello CG, Fasano A, Clemente MG, De Simone C, Silano M, De Vincenzi M, Losito I, Gobbetti M (2006). VSL#3 probiotic preparation has the capacity to hydrolyze gliadin polypeptides responsible for celiac sprue. Biochim Biophys Acta.

[189] Drabinska N, Jarocka-Cyrta E, Markiewicz LH, Krupa-Kozak U (2018). The effect of oligofructose-enriched inulin on faecal bacterial counts and microbiota-associated characteristics in celiac disease children following a gluten-free diet: results of a randomized placebo-controlled trial. Nutrients.

[190] Cammarota G, Ianiro G, Tilg H, Rajilic-Stojanovic M, Kump P, Satokari R, Sokol H, Arkkila P, Pintus C, Hart A, Segal J, Aloi M, Masucci L, Molinaro A, Scaldaferri F, Gasbarrini G, Lopez-Sanroman A, Link A, de Groot P, de Vos WM, Hogenauer C, Malfertheiner P, Mattila E, Milosavljevic T, Nieuwdorp M, Sanguinetti M, Simren M, Gasbarrini A, European FMTWG (2017). European consensus conference on faecal microbiota transplantation in clinical practice. Gut.

[191] Quraishi MN, Widlak M, Bhala N, Moore D, Price M, Sharma N, Iqbal TH (2017). Systematic review with meta-analysis: the efficacy of faecal microbiota transplantation for the treatment of recurrent and refractory clostridium difficile infection. Aliment Pharmacol Ther.

[192] van Beurden YH, van Gils T, van Gils NA, Kassam Z, Mulder CJ, Aparicio-Pages N (2016). Serendipity in refractory celiac disease: full recovery of duodenal villi and clinical symptoms after fecal microbiota transfer. J Gastrointestin Liver Dis.

[193] Sanchez E, Ribes-Koninckx C, Calabuig M, Sanz Y (2012). Intestinal *Staphylococcus* spp. and virulent features associated with coeliac disease. J Clin Pathol.

[194] Roberts SE, Williams JG, Meddings D, Davidson R, Goldacre MJ (2009). Perinatal risk factors and coeliac disease in children and young adults: a record linkage study. Aliment Pharmacol Ther.

[195] Sevelsted A, Stokholm J, Bønnelykke K, Bisgaard H (2014). Cesarean section and chronic immune disorders. Pediatrics.

[196] Adlercreutz EH, Wingren CJ, Vincente RP, Merlo J, Agardh D (2015). Perinatal risk factors increase the risk of being affected by both type 1 diabetes and coeliac disease. Acta Paediatr.

[197] Koletzko S, Lee HS, Beyerlein A, Aronsson CA, Hummel M, Liu E, Simell V, Kurppa K, Lernmark A, Hagopian W, Rewers M, She JX, Simell O, Toppari J, Ziegler AG, Krischer J, Agardh D (2018). cesarean section on the risk of celiac disease in the offspring: the teddy study. J Pediatr Gastroenterol Nutr.

[198] Dydensborg Sander S, Hansen AV, Stordal K, Andersen AN, Murray JA, Husby S (2018). Mode of delivery is not associated with celiac disease. Clin Epidemiol.

[199] Francavilla R, Piccolo M, Francavilla A, Polimeno L, Semeraro F, Cristofori F, Castellaneta S, Barone M, Indrio F, Gobbetti M, De Angelis M (2019). Clinical and microbiological effect of a multispecies probiotic supplementation in celiac patients with persistent ibs-type symptoms: a randomized, double-blind, placebo-controlled. Multicenter Trial J Clin Gastroenterol.

[200] Martinello F, Roman CF, Souza PA (2017). EFFECTS OF PROBIOTIC INTAKE ON INTESTINAL BIFIDOBACTERIA OF CELIAC PATIENTS. Arq Gastroenterol.

[201] Pinto-Sanchez MI, Smecuol EC, Temprano MP, Sugai E, Gonzalez A, Moreno ML, Huang X, Bercik P, Cabanne A, Vazquez H, Niveloni S, Mazure R, Maurino E, Verdu EF, Bai JC (2017). Bifidobacterium infantis NLS super strain reduces the expression of alpha-defensin-5, a marker of innate immunity, in the mucosa of active celiac disease patients. J Clin Gastroenterol.

[202] Harnett J, Myers SP, Rolfe M (2016). Probiotics and the microbiome in celiac disease: a randomised controlled trial. Evid Based Compl Alternat Med.

[203] Uusitalo U, Andren Aronsson C, Liu X, Kurppa K, Yang J, Liu E, Skidmore J, Winkler C, Rewers MJ, Hagopian WA, She JX, Toppari J, Ziegler AG, Akolkar B, Norris JM, Virtanen SM, Krischer JP, Agardh D (2019). Early probiotic supplementation and the risk of celiac disease in children at genetic risk. Nutrients.

[204] Primec M, Klemenak M, Di Gioia D, Aloisio I, Bozzi Cionci N, Quagliariello A, Gorenjak M, Micetic-Turk D, Langerholc T (2019). Clinical intervention using Bifidobacterium strains in celiac disease children reveals novel microbial modulators of TNF-alpha and short-chain fatty acids. Clin Nutr.

[205] Quagliariello A, Aloisio I, Bozzi Cionci N, Luiselli D, D'Auria G, Martinez-Priego L, Perez-Villarroya D, Langerholc T, Primec M, Micetic-Turk D, Di Gioia D (2016). Effect of bifidobacterium breve on the intestinal microbiota of coeliac children on a gluten free diet: a pilot study. Nutrients.

[206] Klemenak M, Dolinsek J, Langerholc T, Di Gioia D, Micetic-Turk D (2015). Administration of bifidobacterium breve decreases the production of tnf-alpha in children with celiac disease. Dig Dis Sci.

